# Functional and Antioxidant Evaluation of Two Ecotypes of Control and Grafted Tree Tomato (*Solanum betaceum)* at Different Altitudes

**DOI:** 10.3390/foods12183494

**Published:** 2023-09-20

**Authors:** Elena Coyago-Cruz, Aida Guachamin, Gabriela Méndez, Melany Moya, Aníbal Martínez, William Viera, Jorge Heredia-Moya, Elena Beltrán, Edwin Vera, Michael Villacís

**Affiliations:** 1 Carrera de Ingeniería en Biotecnología de los Recursos Naturales, Universidad Politécnica Salesiana, Sede Quito, Campus El Girón, Av. 12 de Octubre N2422 y Wilson, Quito 170143, Ecuadorgmendez@ups.edu.ec (G.M.); 2 Facultad de Ciencias Médicas, Carrera de Obstetricia, Universidad Central del Ecuador, Iquique, Luis Sodiro N14-121, Quito 170146, Ecuador; mvmoya@uce.edu.ec; 3 Instituto Nacional de Investigaciones Agropecuarias (INIAP), Programa de Fruticultura, Av. Interoceánica Km15 y Eloy Alfaro, Quito 170518, Ecuador; anibal.martinez@iniap.gob.ec (A.M.); william.viera@iniap.gob.ec (W.V.); 4 Centro de Investigación Biomédica (CENBIO), Facultad de Ciencias de la Salud Eugenio Espejo, Universidad UTE, Quito 170527, Ecuador; jorgeh.heredia@ute.edu.ec; 5 Facultad de Ciencias de la Ingeniería e Industrias, Universidad UTE, Quito 170527, Ecuador; 6 Escuela Politécnica Nacional, Departamento de Ciencias de los Alimentos y Biotecnología, Facultad de Ingeniería Química, Av. 12 de octubre N2422 y Veintimilla, Quito 170524, Ecuador; edwin.vera@epn.edu.ec (E.V.); michael.villacis@epn.edu.ec (M.V.)

**Keywords:** bioactive compound, functional foods, carotenoids, caffeic acid, β-cryptoxanthin, phenolics, vitamin C

## Abstract

Tree tomato (*Solanum betaceum*) is susceptible to nematode attack; for this reason, grafting is used as an alternative to reduce this impact. In this study, the bioactive compounds of the fruit (shell, pulp, and seed jelly) of two tree tomato ecotypes (‘giant orange’ and ‘giant purple’) were evaluated in both control and grafted plants grown at different altitudes (2010–2250, 2260–2500, 2510–2750 and 2760–3000 masl). Commercial quality, vitamin C, organic acids, phenolics, carotenoids and antioxidant activity were determined by microextraction and quantified by liquid chromatography (RRLC) or spectrophotometry (microplate reader). The results showed high concentrations of vitamin C, organic acids and antioxidant activity in the seed jelly, organic acids in the pulp and phenolic compounds, carotenoids, and antioxidant activity in the shell. The main phenolics were ferulic acid, caffeic acid and luteolin, while the main carotenoids were lutein, B-cryptoxanthin and B-carotene. Multivariate analysis showed that tree tomato quality was mainly influenced by altitude and fruit part and that grafting positively affected soluble solids for both ecotypes and all altitudes.

## 1. Introduction

In recent years, there has been increasing interest in nutrition and the consumption of health-promoting foods [1]. Numerous studies have demonstrated the significant health and disease prevention benefits of a balanced diet composed of foods rich in energy, nutrients, and bioactive compounds [2,3]. In this context, *Solanum betaceum* Cav., also known by its synonym *Cyphomandra betacea* (Cav.) Sendtn., and by common names such as ‘buah cinta’, ‘tamarillo’, ‘tree tomato’, ‘wax tomato’ and ‘chilto’, is an exotic crop belonging to the Solanaceae family. It originated in the Andean region of South America and is cultivated in several countries, including Australia, the United States, Colombia, Ecuador, and New Zealand [1,4,5,6,7]. The fruit is ovoid to ellipsoid in shape and varies in size from 4 to 10 cm long and 3 to 5 cm wide, with smooth skin that can vary in colour from yellow to red. The flesh, which is juicy, sweet, and sour, also varies in colour depending on compounds such as chlorophyll, carotenoids, and phenolic compounds such as anthocyanins. The small, dark brown seed is surrounded by gelatinous mucilage [5,8]. 

While several studies have focused on the chemical composition of tree tomato pulp, little attention has been given to the properties of the shell, despite previous research suggesting that different parts of the fruit may have different compositions. Tree tomato pulp has been documented to contain various components such as dietary fibre, starch, soluble sugars (sucrose, glucose and fructose), organic acid (e.g., malic and citric acids), vitamins (e.g., C, B2, B6, and E), phenolic compounds (e.g., anthocyanins, chlorogenic acid, hydroxycinnamic acid), carotenoids (e.g., β-carotene, lutein), terpenoids, steroids, saponins, alkaloids, tannins, proteins, essential amino acids (e.g., glutamic acid, leucine, serine, aspartic acid, and glycine) and minerals (e.g., potassium, phosphorus, calcium, magnesium, iron, copper and zinc) [5,6]. However, the phytochemical composition and fruit quality of tree tomatoes are influenced by various genetic and environmental factors such as light, altitude, temperature, water availability, pre-harvest conditions, rootstock, crop load, fruit position within the tree, cultural practices, and industrial processes [1,4,9,10,11,12,13].

Due to their high content of biologically active metabolites, such as pectin, flavonoids and carotenoids, the tree tomato is on its way to being recognised as a superfood. The pulp of these fruits has shown remarkable health properties, including antioxidant, anti-inflammatory, anticancer, antimicrobial, and anti-obesity activity [14]. These benefits have motivated its use as a natural coagulant in the dairy industry and as an emulsifier and foam stabiliser in various industries. It has also been used as an antioxidant additive in the meat industry and an antinociceptive agent in the pharmaceutical industry [5]. Despite these uses, fruit residues such as shell, seeds and pomace have been little studied and could have significant potential as valuable bioresources for various industrial applications [15]. 

On the other hand, the exponential growth of the world’s population has led to increased food insecurity and intensified food production [16]. Unfortunately, some agricultural practices that meet the growing demand have proved inadequate, leading to crop failure and increased pest populations. In particular, tree tomato crops are particularly susceptible to diseases caused by viruses, bacteria, nematodes and fungi [17]. To address these challenges, rootstock grafting has been highlighted as an important strategy to control soil-borne pests and diseases in fruit trees [18], and studies have shown that grafting improves yield, fruit weight, mineral content, and sensory characteristics [19]. Grafting tree tomatoes to *Nicotiana glauca* has reduced susceptibility to soil-borne microorganisms (e.g., *Meloidogyne incognita* and *Fusarium solani*), prolonging the plant´s productive life [20]. 

In this context, there is a significant lack of literature on the bioactive compounds in the different parts of the fruit of grafted tree tomatoes grown at different altitudes. Therefore, this study aimed to evaluate the bioactive compounds of the fruit (shell, pulp, and seed jelly) of two ecotypes of tree tomatoes (‘giant orange’ and ‘giant purple’), considering control and grafted plants and their development at different growing altitudes (2010–2250, 2260–2500, 2510–2750 and 2760–3000 masl). The results of this study will provide valuable information as a starting point for understanding the influence of tree tomato rootstock on bioactive compounds, contributing to the promotion of more effective agricultural practices and the improvement of the quality of the food produced.

## 2. Materials and Methods

### 2.1. Chemicals

Analytical-grade acetone (CAS 67-64-1), HPLC-grade acetonitrile (CAS 75-05-8), HPLC-grade ethanol (CAS 64-17-5), HPLC-grade ethyl acetate (CAS 141-78-6), HPLC-grade methanol (CAS 67-56-1), and analytical-grade trichloromethane (CAS 67-66-3) were purchased from Fisher Chemical (Fischer Scientific Inc., Madrid, Spain). ABTS (2,2’-azino-bis-(3-ethylbenzothiazoline-6-sulphonic acid)) (CAS 30931-67-0), *DL*-homocysteine (CAS 454-29-5), DPPH (2,2-Diphenyl-1-picrylhydrazyl) (CAS 1898-66-4), formic acid (CAS 64-18-6), metaphosphoric acid (CAS 37267-86-0), n-acetyl-n,n,n-trimethyl ammonium bromide (CAS 57-09-0), potassium persulphate (CAS 7727-21-1), monobasic potassium phosphate (CAS 7778-77-0), and analytical-grade sulphuric acid (7664-93-9) were purchased from Sigma (Merck, Darmstadt, Germany). Analytical-grade hydrochloric acid (CAS 7647-01-0) was purchased from Labscan (RCI Labscan group, Dublin, Republic of Ireland). Deionised water was purified using a NANOpureDiamond^TM^ system (Barnsted Inc. Dubuque, IO, USA).

Vitamin C standards, including *L*-(+)-ascorbic acid 99.8% (CAS 50-81-7); organic acid standards, such as citric acid 100.8% (CAS 77-92-9), malic acid 99.0% (CAS 97-67-6), *L*-(+)-tartaric acid 99.5% (87-69-4); phenolics standard, such as caffeic acid 98.0%(CAS 331-39-5), chlorogenic acid 95.0% (CAS 327-97-9), chrysin 97.0% (CAS 480-40-0), *p*-coumaric acid 98.0% (CAS 501-98-4), *m*-coumaric acid 99.0% (CAS 588-30-7), *o*-coumaric acid 97.0% (CAS 614-60-8), ferulic acid 100.0% (CAS 1135-24-6), gallic acid 100.0% (CAS 149-91-7), *p*-hydroxybenzoic acid (CAS 99-96-7), 3-hydroxybenzoic acid 99.0% (CAS 99-06-3), 2,5-dihydroxybenzoic acid 98.0% (CAS 490-79-9), kaempferol 97.0% (CAS 520-18-3), luteolin 98% (CAS 491-70-3), naringin 95.0% (CAS 10236-47-2), quercetin 95.0% (CAS 849061-97-8), rutin 94.0%(CAS 153-18-4), shikimic acid 99.0% (CAS 138-59-0), syringic acid 95.0% (CAS 530-57-4), vanillic acid 97.0% (CAS 121-34-6); carotenoids standard, such as β-carotene 93.0% (CAS 7235-40-7), β-cryptoxanthin 97.0% (CAS 472-70-8), lutein (CAS 127-40-2), lycopene (CAS 502-65-8)and zeaxanthin (CAS 144-68-3); and antioxidant activity standard, Trolox 98% (CAS 53188-07-1), were purchased from Sigma (Merck, Darmstadt, Germany). 

### 2.2. Plant Material and Soil Analysis

This study included mature fruits of two ecotypes: ‘giant orange’ (GOE), characterised by its red external colour and orange internal colour, and ‘giant purple’ (GPE), with red exterior colour and purple interior colour. To evaluate the influence of grafting, plants grafted on ‘tabaquillo’ (*Nicotiana glauca* G.) were used, while non-grafted plants were used as a control group. In addition, to evaluate the effect of altitude, plants were grown at different altitudes ranging from 2010 to 3000 m above sea level (masl), divided into 240-m intervals (2010–2250, 2260–2500, 2510–2750 and 2760–3000 masl). In addition, this study considered the analysis of the different parts of the fruit, including the shell, pulp, and seed jelly. Consequently, an experimental plot was established for each altitude studied, resulting in seven experimental plots of approximately 100 plants each, giving a total of 700 tree tomato plants for this study.

The tree tomato plants used in this study were grown according to the agronomic practices recommended by the National Institute of Agricultural Research (INIAP). They were developed in the open air on sandy or loamy soils with moderate organic matter. The location selected for cultivation was Tungurahua, Ecuador, recognised as the main production area, with a temperature and generally dry climate (Table 1). Plants were spaced 1 m apart and 1.5 m between rows. To ensure optimum development, irrigation was applied every 15 days, providing an average of 5.3 litres of water per plant. In addition, regular nutrient applications were made according to the soil analysis results (Table 2 and Table 3) [21].

Soil analysis of the experimental plots was carried out at a depth of 30 cm and included several important parameters. The soil type was determined and classified as sand, clay, and silt. In addition, pH was evaluated with 1:2.5 of water (pHs), electrical conductivity with sodium: saturated paste (EC), and organic matter (OM) with the Walkley and Black method [22] were evaluated. Colourimetric analysis was used to determine the ammonium (NH_4_)^+^ and nitrate (NO_3_)^−^ ions, while the PEE/ABL/01 method was used to determine the ions phosphorus (P^3−^), potassium (K^+^), calcium (Ca^2+^), magnesium (Mg^2+^), manganese (Mn^2+^), iron (Fe^2+^ and Fe^3+^), sodium (Na^+^), copper (Cu^2+^), zinc (Zn^2+^), which was validated by AGROBIOLAB limited company. Finally, the modified Olsen method was used to evaluate aluminium (Al^3+^), while the colourimetric method was used to analyse boron (B), and sulphur ion (SO_4_)^2−^ with a 95% confidence level [23]. 

Chemical fertilisation was applied every three months with a combination of YaraMila COMPLEX ^®^ plus and calcium nitrate at 100 g and 50 g per plant, respectively. In addition, a single dose of 1 kg of chicken and cow manure was applied per plant. To ensure plant health, monthly phytosanitary controls were carried out with a rotation of agrochemicals, including sulphur 0.1%, penconazole 0.1%, captan 0.1%, fosetyl aluminium 0.1%, cyclohexanone plus dimethoate 0.1%, diflubenzuron 0.1%, agricultural oil 0.5%, and neutralised Bordeaux broth 0.5%. 

To avoid the edge effect, 20 trees in the middle of each plot were randomly selected. Three fruits were harvested from each plant, with an average of two years of vegetative development. In each experimental plot, sixty fruits were collected under optimal visual conditions, with a colour range of 5 to 6, corresponding to ripe fruit, according to the specifications of the NTE INEN 1909 standard scale “Frutas frescas. Tomate de árbol. Requisitos” [24]. These fruits were selected according to the protocol based on NTE INEN 1750:1994, “Hortalizas y frutas frescas. Muestreo”, which recommends randomly selecting 2 kg of fruit for chemical analysis [25]. All fruits were harvested simultaneously in July 2020, during the dry season. To preserve the quality of the harvested fruits, they were transported in paper-protected containers and processed immediately. Forty fruits were randomly selected from each group of harvested tree tomatoes to assess their marketable quality, while the remaining 20 were used to separate shell, pulp, and seed jelly. Each fraction was individually frozen at −80 °C and freeze-dried in a Christ Alpha 1–4 LDplus equipment (Martin Gefriertrocknungsanlagen GmbH, Osterode am Harz, Germany). The freeze-dried samples were stored in hermetically sealed dark glass bottles under a nitrogen atmosphere and kept in a freezer at −21 °C until analysis.

### 2.3. Evaluation of Commercial Quality

The commercial quality of fresh tree tomatoes was assessed according to NTE INEN 1909:2016 [24]. CIELAB colour measurements (L*, C_ab_* and h_ab_) were performed on whole fruits using a Konica Minolta Chroma Meter CR-400 tristimulus colourimeter (Konica Minolta Sensing Americas, Ramsey, NJ, USA) [26] and equatorial (ED) and longitudinal (LD) diameters were measured using a Titan 23,175 electronic digital calliper (Titan, Rancho Cucamonga, CA, USA). In addition, the weight of the fruit and its components (shell, pulp, and seed jelly) was recorded using a Mettler Toledo ML204T/00 analytical balance (Mettler Toledo, Greifensee, Switzerland). The edible part (shell and seed jelly) was then ground in a mortar and pestle until a homogeneous paste was obtained, which was used to quantify the soluble solids (°Brix) using a refractometer (Boeco, Hamburg, Germany). Likewise, the pH was measured with a digital Seven multi-pHmeter-conductimeter (Mettler Toledo, Switzerland), and the total titratable acidity was determined according to NTE INEN-ISO 750:2013, expressed in grams of citric acid per 100 mL [27]. Moisture was quantified in a Be20 air circulation oven (Memmert GmbH Co.KG, Schwabach, Germany) at 70 °C, while ash content was quantified using a Thermolvne muffle (Thermo Fisher Scientific, Waltham, MA, USA) at 550 °C. Finally, the maturity index was calculated by dividing the total titratable acid by the soluble solids obtained.

### 2.4. Bioactive Compounds Quantification

#### 2.4.1. Vitamin C Quantification

The quantification of vitamin C or ascorbic acid followed the NSAI method with some modifications [28]. The extraction process was performed in triplicate. Thus, one gram of freeze-dried powder was extracted using 2.5 mL of 0.2% *DL*-homocysteine, and 15 mL of 3% metaphosphoric acid. The mixture was vortexed and sonicated in a Fisher Scientific FS60 ultrasonic bath (Fisher Scientific, Hampton, NH, USA) for 7 min, and the volume was adjusted to 25 mL with deionised water. This solution was centrifuged at 13,171× *g*, 4 °C for 5 min using a MiniSpin series microcentrifuge (Eppendorf, Hamburg, Germany). The supernatant was filtered through a 0.45 µm PVDF (Polyvinylidene fluoride) filter. The filtrate was quantified using an Agilent 1200 series Rapid resolution liquid chromatography (RRLC) system (Agilent Technologies, Santa Clara, CA, USA) equipped with a DAD–UV–VIS detector set at 244 nm, and an HPLC column Zorbax Eclipse, XDB- C_18_, 80 Å (pore size) (4.6 × 50 mm column size, 1.8 µm particle size, 600 bar pressure limit) (Agilent Technologies, USA). The column temperature was maintained at 30 °C, and the flow rate under isocratic conditions was 1 mL/min. The mobile phase consisted of 90% monobasic potassium phosphate 1.5% and 10% methanolic solution of n-acetyl-n,n,n-trimethyl ammonium bromide 1.8%. The total run time was 20 min, and the injection volume was 20 µL. The analysis was performed in duplicate, and the data acquisition and chromatogram processing were carried out using Open lab ChemStation (version 2.15.26) software. Vitamin C was identified by comparing retention times, UV–vis spectra, and an internal standard. Chromatograms were monitored at 244 nm, and quantification was done using external calibration curves that included a 1 mg/mL concentration of *L*-(+)-ascorbic acid standard with different injection volumes (3, 5, 10, 15, and 20 µL). The limits of detection (LOD) and quantification (LOQ) were determined based on a signal-to-noise ratio (S/N) of 3 and 10, respectively, using the relative standard deviation of blank analytical values calculated from the calibration curve. The LOD and LOQ values were 0.20 ppm and 0.65 ppm, respectively. Vitamin C was expressed as milligrams per 100 grams of dry weight (mg/100 g DW).

#### 2.4.2. Analysis of Organic Acid

The quantification of individual organic acids followed the method described by Macrae with some modifications [29]. The extraction process was performed in triplicate. Thus, 40 mg of freeze-dried powder was extracted with 1.5 mL of 0.02 N sulphuric acid containing 0.05% metaphosphoric acid and 0.02% *DL*-homocysteine. The mixture was vortexed and sonicated in an ultrasound bath for 3 min, and the volume was adjusted to 2 mL with deionised water. This solution was then centrifuged at 13,171× *g* at 4 °C for 5 min, and the supernatant was filtered through a 0.45 µm PVDF filter. The filtrate was quantified using an RRLC 1200 model equipped with a DAD–UV–VIS detector set at 210 nm and a YMC-Triart C18 column (150 × 4.6 mm column size, 3 µm particle size, 12 nm pore size, 400 bar pressure limit) (YMC Europe GmbH, Dinslaken, Germany). The column temperature was maintained at 30 °C, and the flow rate under isocratic conditions was 1 mL/min. The mobile phase consisted of 0.027% sulphuric acid, and the total run time was 30 min with an injection volume of 20 µL. Individual organic acid identification was done by comparing retention times, UV–vis spectra, and an internal standard. Chromatograms were monitored at 210 nm, and the analysis was performed in duplicate using Open lab ChemStation software for data acquisition and chromatogram processing. The quantification of organic acids was conducted using external calibration curves, which included a 100 mg/mL concentration of citric, malic, and *L*-(+)-tartaric acid standards prepared and quantified separately with injection volumes of 3, 5, 10, 15 and 20 µL. The detection (LOD) and quantification (LOQ) limits were determined as 0.02 and 0.06 ppm for citric acid, 0.07 and 0.23 ppm for malic acid, and 0.02 and 0.07 ppm for tartaric acid, respectively. The total organic acid content was calculated by summing up all individual compounds, and the organic acid content was expressed as grams per 100 g of dry weight (g/100 g DW).

#### 2.4.3. Phenolics Quantification

Phenolics were extracted using the microextraction method described by Meléndez-Martínez et al. [30], with the extraction process performed in triplicate. 40 mg of freeze-dried powder was mixed with 1000 µL of methanol acidified to 80% with HCl 0.1%. The mixture was vortexed, sonicated for 2 min and then centrifuged at 13,171× *g* and 4 °C for 5 min. The supernatant was collected, and the solid residue was subjected to two additional extractions using 500 µL of acidified methanol. The resulting supernatants were combined, filtered through a 0.45 µm PVDF filter, and quantified using an RRLC 1200 model equipped with a DAD–UV–VIS detector set between 220 and 500 nm and a Zorbax Eclipse Plus C18 column (4.6 × 150 mm column size, 5 µm particle size) (Agilent Technologies, Santa Clara, CA, USA). The column was maintained at 30 °C, and the flow rate was set at 1 mL/min. The mobile phase consisted of 0.01% formic acid in water (solvent A) and acetonitrile (solvent B), with a linear gradient elution as follows: 100% A at 0 min; 95% A + 5% B at 5 min; 50% A + 50% B at 20 min; washing and re-balancing of the column at 22 min. The injection volume was 10 µL, and the analysis was conducted in duplicate using Open lab ChemStation software for data acquisition and chromatogram processing. The identification of phenolic compounds was based on comparing retention times and UV–vis spectra in the range of 250 to 750 nm with available standards. Chromatograms were monitored at 280, 320, and 370 nm. The quantification of phenolic compounds was carried out using external calibration curves, which included a 1 mg/mL concentration of caffeic acid, chlorogenic acid, chrysin, *p*-coumaric acid, *m*-coumaric acid, *o*-coumaric acid, ferulic acid, gallic acid, *p*-hydroxybenzoic acid, 3-hydroxybenzoic acid, 2,5-dihydroxybenzoic acid, kaempferol, luteolin, naringin, quercetin, rutin, shikimic acid, syringic acid, vanillic acid standards. These standards were prepared and quantified separately with 3, 5, 10, 15 and 20 µL injection volumes. LOD and LOQ ranged from 0.006 to 0.014 µg in chlorogenic acid and from 0.012 to 0.041 µg for *p*-hydroxybenzoic acid, respectively. The phenol concentration was expressed as milligrams per 100 grams of dry weight (mg/100 g DW), and the total phenolics were calculated by summing up all individual compounds.

#### 2.4.4. Analysis of Carotenoids

Carotenoids were extracted and analysed following the method described by Coyago-Cruz et al. [31]. The extraction process was performed in triplicate. Specifically, 20 mg of freeze-dried powder was extracted using a mixture of 250 µL of methanol, 500 µL of trichloromethane, and 250 µL of deionised water. The mixture was vortexed, sonicated for 2 min, then centrifuged at 13,171× *g*, 4 °C for 5 min. The supernatant was collected, while the solid residue underwent additional extractions until the colour was removed entirely. The coloured organic fractions were evaporated to dryness using a Buchi TM R-100 rotary evaporator (Fisher Scientific, USA), keeping the temperature below 30 °C. The resulting dried extract was then dissolved in 40 µL of ethyl acetate before injection into the RRLC 1200 system, equipped with a DAD–UV–Vis detector and a C18 Poroshell 120 column (2.7 µm particle size, 5 cm × 4.6 mm column size) (Agilent Technologies, USA). The column was maintained at 30 °C, and the flow rate was set to 1 mL/min. The mobile phase consisted of acetonitrile (solvent A), methanol (solvent B), and ethyl acetate (solvent C) with the following linear gradient elution: 85% A + 15% B at 0 min; 60% A + 20% B + 20% C at 5 min; 60% A + 20% B + 20% C at 7 min; 85% A + 15% B at 9 min; 85% A + 15% B at 12 min. The injection volume was 10 µL, and the analysis was conducted in duplicate using Open lab ChemStation software for data acquisition and chromatogram processing. The identification of carotenoids was accomplished by comparing retention times and UV–vis spectra. Chromatograms were monitored at 285, 350, and 450 nm. Quantification of carotenoids was performed using external calibration curves, which included a 1 mg/mL concentration of β-carotene, β-cryptoxanthin, lutein, lycopene, and zeaxanthin standards. These standards were prepared and quantified separately with injection volumes of 3, 5, 10, 15 y 20 µL. LOD and LOQ ranged from 0.002 to 0.007 µg in phytoene and from 0.070 to 0.232 µg for lycopene, respectively. Carotenoid concentrations were expressed as milligrams per 100 grams of dry weight (mg/100 g DW), and the total carotenoids were calculated by summing up all individual compounds.

### 2.5. Antioxidant Activity Determination

#### 2.5.1. Antioxidant Activity by ABTS (2,2′-azino-bis-(3-ethylbenzothiazoline-6-sulphonic acid) Assay

The extraction and measurement of antioxidant activity were carried out in triplicate. For the liquid extract, 0.1 g of freeze-dried powder was mixed with 800 µL of a 50:50 methanol: water solution. The mixture was sonicated for 2 min, followed by centrifugation at 13,171× *g* and 4 °C for 3 min. The resulting supernatant was separated while the solid residue was subjected to extraction with 800 µL of acetone: deionised water solution (56:24). This extract was also sonicated and centrifuged as described earlier. The combined supernatant was then refrigerated until further quantification.

To generate the ABTS^•+^ radical, a 1:1 solution of 7 mM ABTS and 2.45 mM potassium persulphate was prepared and allowed to stand in the dark for 16 h. After the specified incubation period, the ABTS^•+^ radical solution was diluted approximately 1 to 10 with absolute ethanol until an absorbance of 0.7 at 734 nm was obtained [32,33]. A calibration curve was established using a 2.5 nM Trolox standard in ethanol, diluting 0, 12.5, 25, 50 and 75 µL in 300 µL. For sample quantification, 20 µL of either the standard or liquid extract was added to a 96-well VWR Tissue culture plate (Corning, Glendale, AZ, USA), along with 280 µL of ABTS^•+^ radical solutions [34]. The absorbance was measured at 734 nm using a Thermo Scientific Multiskan GO microplate reader spectrophotometer (Agilent Scientific Instruments, Santa Clara, CA, USA). Antioxidant activity was expressed as millimolar Trolox equivalents per gram of dry weight (µmol TE/g DW). 

#### 2.5.2. Antioxidant Activity by DPPH (2,2-Diphenyl-1-Picrylhydrazyl) Method

The DPPH method’s antioxidant activity was determined following the procedure outlined by Pires et al. with some modifications [35]. The extraction and measurement for antioxidant activity were conducted in triplicate. For the liquid extract, 20 mg of freeze-dried powder was mixed with 2 mL of methanol. The mixture was sonicated for 3 min, followed by centrifugation at 13,171× *g*, and 4 °C for 3 min to collect the supernatant. A standard stock solution was prepared by dissolving a 1 mg/mL *L*-(+)-ascorbic acid standard in methanol and diluting it to 50%. The calibration curve consisted of five concentration points ranging from 0 to 6 mg/mL. To prepare the DPPH radical solution, 10 mg of DPPH was dissolved in 50 mL of methanol. The reaction was initiated by mixing 20 µL of the standard or liquid extract with 280 µL of the DPPH- radical solution in a 96-well VWR Tissue culture plate. Separate wells containing 300 µL of methanol and 300 µL of the DPPH^−^ radical solution were used as blanks. The absorbance was measured after 30 min of continuous shaking in the dark using a 4310 Shaker Orbital (Fisher Scientific, USA) and a BioTek H1 spectrophotometer (Scientific Instruments, Santa Clara, CA, USA) at 560 nm. The antioxidant activity was expressed in millimolar ascorbic acid equivalents per gram of dry weight (µmol AAE/g DW).

### 2.6. Statistical Analysis

Statistical analyses were performed using Statgraphics Centurion XVII, SigmaPlot (version 14.0), and RStudio (version 4.2.3). Results are presented as mean ± standard deviation. Simple and factorial analyses of variance (ANOVA) were used to investigate the effects of ecotype, grafting, altitude, and fruit part. The mean was separated using Tukey’s test with a significance level of 0.01 to identify significant differences. Pearson correlations were used with a 99% confidence level to identify possible associations. In addition, principal components analysis (PCA) was used to determine the most influential variables. 

## 3. Results and Discussion

### 3.1. Soil Analysis

Table 2 and Table 3 present the soil analysis results in the plantations of the ‘giant orange’ (GOE) and ‘giant purple’ (GPE) tree tomato ecotypes, respectively. The soil composition of the plantations, according to the United States Department of Agriculture (USDA) texture triangle [36], was 58.4% sand, 15.2% clay, and 26.4% silt. In addition, soil pH ranged from 5.3 (acidic) to 6.9 (near neutral), electrical conductivity (EC) from 0.9 to 9.1 mmhos/cm and organic matter content from 1.0 to 5.8%. Concerning nutrients, variable concentrations were found, such as (NH_4_)^+^ between 25.4 and 116.1 ppm, (NO_3_)^−^ between 56.1 to 463.4 ppm, P^3−^ between 55.4 and 625.0 ppm, K^+^ between 0.5 and 4.2 meq/100 mL, Ca^2+^ between 7.0 and 17.6 meq/100 m, Mg^2+^ between 1.9 and 6.9 meq/100 mL, Mn^2+^ between 9.8 and 92.4 ppm, and Fe^2+,3+^ between 22.6 and 2500 ppm. In addition, Na^+^ was found to range from 0.1 to 0.4 meq/100 mL, Al^3+^ from 0.3 to 0.7 meq/100 mL, Cu^2+^ from 3.6 to 11.1 ppm, Zn^2+^ from 2.4 to 33.4 ppm, B from 1.8 to 8.4 ppm and (SO_4_)^2−^ from 8.1 to 79.8 ppm in the soil. 

Statistical analysis of the soil showed, in some cases no significant difference between the two ecotypes, indicating a similarity of soil characteristics in the study area and helping to reduce the study variables. Soil pH was found to be within the agronomic recommendations for the crop [37], indicating the soil’s ability of the soil to retain nutrients such as calcium, magnesium and potassium, indicating a lower frequency of fertiliser application. However, an accumulation of manganese and a decrease in calcium were observed in soils with a pH below 5.5 [36].

In general, the soils of the orchards studied showed characteristics favourable to tree tomato cultivation and corresponded to soils suitable for agriculture, as reported in other studies [36,38,39]. The cation exchange capacity, which measures the ability of the soil to retain and store elements such as calcium, magnesium, and potassium, showed medium and high values, indicating less frequent application of composts at higher doses. This characteristic is reflected in the levels of macronutrients such as (NO_3_)^−^, P^3−^, K^+^ and Ca^2+^ as well as micronutrients such as Cu^2+^, Fe^2+^, Fe^3+^, Mn^2+^ y Zn^2+^, which were excessive in most of the experimental soils, resulting in low availability of P^3−^ due to the high Ca^2+^ content. In this study, it was observed that nitrate (NO_3_)^−^ was 3.5-fold higher than ammonium (NH_4_)^+^ in nitrate, as suggested by other authors [23], which allows plants to absorb nitrogen from the soil.

### 3.2. Evaluation of Commercial Quality

**Figure 1:**
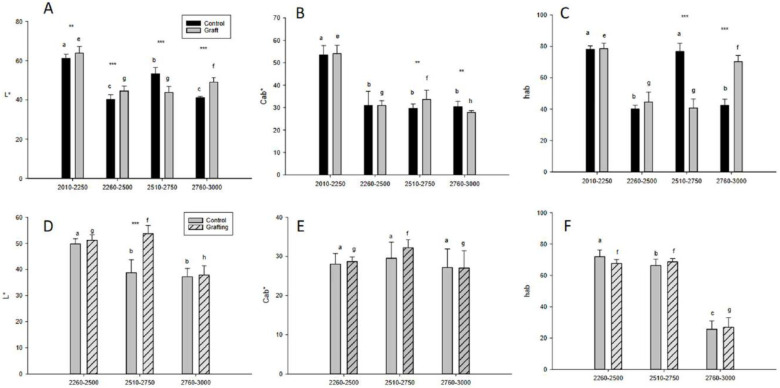
CIELAB colour coordinates of ‘giant orange’ (GOE) (**A**–**C**) and ‘giant purple’ (GPE) (**D**–**F**) tree tomato ecotypes grown at different altitudes. The asterisks show statistical differences between the control and graft, and non-significant is not shown, ** *p* < 0.01, *** *p* < 0.001 and different lowercase letters indicate a significant difference between altitude ranges in the same treatment (control or graft).

Data on commercial quality parameters of the tree tomato ecotypes ‘giant orange’ (GOE) and ‘giant purple’ (GPE), including fruit weight, pulp weight, seed jelly weight, equatorial and longitudinal diameters, pH, % total titratable acid, soluble solids,% humidity,% ash, and maturity index are summarised in Table 4. In addition, the fruit colour (L*, C_ab_*, and h_ab_) is shown in Figure 1. 

#### 3.2.1. Fruit Weight

Fruit weights of tree tomatoes of the ‘giant orange’ ecotype (GOE) ranged from 75.9 to 160.4 g, while those of the ‘giant purple’ ecotype (GPE) ranged from 105.7 to 144.4 g (Table 4). These results agree with previous studies that reported similar fruit weight values for both ecotypes (102.5 g and 92.6 g for GOE and GPE, respectively) [4], as well as with a literature review on tree tomato that indicated a weight range between 30.0 and 160.0 g [40]. 

Regarding grafting, it was observed that the fruit weight of GOE tree tomato in control plants ranged from 75.9 to 146.0 g. While in grafted plants, it ranged from 127.0 to 160.4 g. On the other hand, in GPE, the weight of tree tomatoes in control plants ranged from 105.7 to 144.4 g. Grafting had a significant effect on fruit weight in both ecotypes, resulting in an increase. However, there were exceptions, such as at altitudes of 2010–2250 masl and 2260–2500 masl in GOE and 2510–2750 masl in GPE, where the relationship between the control and grafting groups showed no significant difference in fruit weight. These results were in line with other studies that reported an increase in fruit weight in grafted plants of *Pistacia atlantica* [19]. 

Regarding fruit parts, it was observed that the pulp weight of GOE ranged from 49.5 to 93.7 g, while that of GPE ranged from 62.4 to 85.7 g. The seed jelly weight of GOE ranged from 17.4 to 57.4 g, while GPE ranged from 28.9 to 62.7 g. At the same time, it is interesting to note that in the control group, higher values of pulp weight and seed jelly were found in GOE compared to data reported in other studies for the edible fraction of GOE (pulp and seed jelly), which ranged from 71.9 g to 78.5 g in crops grown in the regions of Pelileo-Tungurahua (2572 masl) and Chaltura-Imbabura (2351 masl), respectively [4]. This difference suggests that the agronomic practices used in this study may have influenced the development of GOE fruits with higher pulp and seed jelly content, as indicated by other studies [3]. 

Regarding altitude, it was observed that the highest values for fruit weight, pulp and seed jelly were found at altitudes of 2510–2750 masl for GOE and 2260–2500 masl for GPE. At the same time, altitude showed a significant difference in fruit weight in the control and the grafted group. These results are consistent with previous reports indicating that tree tomato thrives at a broad range of altitudes in Ecuador, between 1525 and 3050 masl [40]. This suggests that the mid-range altitudes found in this study may contribute to larger fruit size, as fruits grown at higher altitudes have a higher transpiration rate, resulting in a longer flow of water and nutrients, and lower fruit weight [41]. This could explain the higher values observed at higher altitudes, such as between 2760 and 3000 masl in the GOE. 

#### 3.2.2. Fruit Size

The equatorial diameter (ED) of GOE ranged from 4.7 to 6.9 cm, while that of GPE ranged from 5.3 to 6.0 cm (Table 4). The longitudinal diameter (LD) of GOE ranged from 5.8 to 8.1 cm, while that of GPE ranged from 7.0 to 7.9 cm. These results indicate that the evaluated ecotypes presented fruits with considerably acceptable dimensions for marketing, as indicated by the requirements of INEN 1909:2015, which establishes an ED ≥ 5.5 cm and an LD ≥ 7.0 cm [24]. On the other hand, in the case of GPE, LD showed lower values compared to data reported by other authors for fruits grown in Malaysia, which ranged from 9 to 12 cm [7]. This difference in size could be related to the specific climatic conditions of the study sites, as suggested by other researchers [3,26]. For example, the average temperature in Tungurahua, Ecuador, where this study was conducted, was 13 °C, while in Malaysia, it was 27 °C.

Regarding grafting, it was observed that the ED of GOE tree tomato fruit on control plants ranged from 4.7 to 6.2 cm, while on grafted plants, it ranged from 5.8 to 6.9 cm. On the other hand, in GPE, the ED of tree tomato ranged from 5.3 to 5.8 cm in control plants and from 5.7 to 6.0 cm in grafted plants. The LD for GOE ranged from 6.1 to 7.3 cm in the control group and from 5.8 to 8.1 cm in the grafted group. For GPE, the LD ranged from 7.1 to 7.9 cm in the control group and from 7.0 to 7.5 cm in the transplanted group. In this regard, control and grafting showed a significant relationship on equatorial and longitudinal diameters, with an increase in size in most cases for both ecotypes, except for LD and ED at heights of 2010–2250 masl for GOE, and LD at heights of 2510–2750 masl and 2760–3000 masl for GPE. In addition, ED and LD showed a relationship with data from a literature review on control tree tomatoes, which showed a range between 4 and 10 cm in length and between 3 and 6 cm in cross-section [40]. 

Regarding altitude, it was observed that the highest ED values in GOE were found at an altitude of 2260–2500 masl in both control and grafted plants. On the other hand, in GPE, the highest ED values were found at altitudes of 2760–3000 masl and 2260–2500 masl in control and grafted plants, respectively. In turn, the highest LD values in GOE were found at 2010–2250 masl and 2260–2500 masl in control plants, while in grafted plants, the highest LD value was observed at 2760–3000 masl. In the case of GPE, the highest LD values occurred at 2760–3000 masl and 2260–2500 masl in control and grafted plants, respectively. At the same time, height showed a significant difference in fruit size in the control and grafted groups in both ecotypes. Furthermore, previous research has documented that the weight and size of *S. betaceum* fruits grown in Ecuador and Spain were influenced by the different altitudes at which the study samples were found [10]. 

#### 3.2.3. Fruit pH

The pH of tree tomato fruits of the ‘giant orange’ ecotype (GOE) ranged from 3.2 to 6.0, while that of the fruits of the ‘giant purple’ ecotype (GPE) ranged from 3.4 to 4.4 (Table 4). These results were consistent with other studies that reported pH values of 3.8 for GOE and 3.5 for GPE [42], as well as research indicating that tree tomatoes with low yellow to orange-red shades tend to be relatively acidic, with pH values between 3.7 and 3.8 [9].

Regarding grafting, it was observed that the pH of GOE tree tomato in control plants ranged from 3.3 (to 5.8, while in grafted plants, it ranged from 3.2 to 6.0. On the other hand, in GPE, the pH of tree tomato in control plants ranged from 3.4 to 4.4, while in grafted plants, it ranged from 3.5 to 4.4. It is important to note that the relationship between control and grafting significantly affected pH in all the cases for both ecotypes. This indicates that the grafting process influences the acidity characteristics of tree tomato fruits. These results highlight the importance of considering grafting as an agronomic practice that can modulate the pH of the fruit, thus influencing its flavour and quality.

Regarding altitude, it was observed that in GOE, the highest pH values were found at altitudes of 2210–2250 masl in both control and grafted plants. On the other hand, in GPE, the highest pH values were observed at altitudes of 2510–2750 masl in the control plants, while in the grafted plants these higher values occurred at altitudes of 2760–3000 masl.

#### 3.2.4. Total Titratable Acidity

The total titratable acidity (TTA) of GOE ranged from 0.8 to 1.9% while that of the fruits of GPE ranged from 0.6 to 1.4% (Table 4). These results were within the titratable acidity requirements for tree tomato consumption, which set a maximum of 2% citric acid [24]. The ATT values obtained in this study were similar to those reported by other authors [4], who found ATT values between 1.1% and 1.7% for the orange ecotype and other tree tomato segregants. It also agrees with previous research that reported a value of 1.7% for the purple ecotype at an altitude of 2340 m asl [43]. However, there were slight differences with other studies where ATT values ranged from 1.2 to 1.8% [5].

Regarding grafting, it was observed that TTA of GOE ranged from 1.1 to 1.4% in control group plants, while in grafted plants this value ranged from 0.8 to 1.9%. In the case of GPE, the ATT values ranged from 0.8 to 1.3% in control plants and from 0.6 to 1.4% in grafted plants. It is important to note that in the case of GPE, grafting did not induce significant changes in ATT of tree tomato fruits.

Regarding altitude, it was observed that the highest levels of total titratable acidity were found in the plants of the grafted group, specifically at altitudes of 2260–2500 masl for GOE and 2760–3000 masl for GPE. Altitude showed a significant difference in the total titratable fruit acid in control and grafted in the two ecotypes.

#### 3.2.5. Soluble Solids

The soluble solids (SS) of tree tomato fruits of the ‘giant orange’ ecotype (GOE) ranged from 8.2 to 12.5 °Brix, while that of the fruits of the ‘giant purple’ ecotype (GPE) ranged from 8.7 to 11.1 °Brix (Table 4). The results of this study are consistent with other studies reporting a range of 10.0 to 13.5 °Brix for GOE [9].

Regarding grafting, it was observed that in GOE, SS of tree tomato in control plants ranged from 8.2 to 10.7 °Brix, while in grafted plants, it ranged from 9.4 to 12.5 °Brix. On the other hand, in GPE, the soluble solids of tree tomatoes ranged from 8.7 to 9.9 °Brix in control plants and from 10.1 to 11.1 °Brix in grafted plants. In turn, grafting showed a significant effect on SS content was observed in all cases for both ecotypes. Grafting resulted in an increase in the amount of SS in the fruit at all altitudes. These results agree with authors who reported values from 10.0 to 12 °Brix in orange tree tomato segregants grafted on *N. glauca* and grown at 2340 masl [11,43]. These significant differences can be attributed to the accumulation of reducing sugars during fruit ripening, which are the main components of SS. In addition, SS values may vary according to cultivar, as suggested by other authors [40]. In turn, Alvarado et al. [1] also pointed out that grafting has been developed to increase the sugar content in fruits. In this sense, the influence of grafting on fruit quality is highlighted, with sweeter fruits reported than those grown using traditional techniques.

Regarding altitude, it was observed that the highest values of soluble solids were found in grafted plants at an altitude of 2010–2250 masl in GOE and 2260–2500 masl in GPE. In turn, the altitude showed a significant difference in the soluble solids content of the fruit in plants of the control group and plants of the grafted group in the two ecotypes. The SS results obtained for GOE differ from those of other authors [44], who observed higher SS levels in fruit grown at higher altitudes due to more significant solar radiation, lower temperatures and relative humidity. 

#### 3.2.6. Moisture

The moisture content of tree tomato fruits of GOE ranged from 79.2 to 98.3%, while that of GPE ranged from 77.5 to 87.8% (Table 4). These results agreed with other authors who reported a moisture value of 85.2% for GPE [7] and a range of 86.0 to 92.0% in puree (pulp and seed jelly) of yellow and giant purple tomatoes [5].

Regarding grafting, it was observed that in GOE, tree tomato moisture in control plants ranged from 79.2 to 98.3%, while in grafted plants it ranged from 79.3 to 86.1%. On the other hand, at GPE, the moisture content of tree tomatoes in control plants ranged from 80.0 to 87.8%, while in grafted plants it ranged from 77.5 to 84.9%. In most cases, the ratio between the control and graft groups significantly affected the moisture percentage, except for the GPE at altitudes of 2510–2750 masl and 2760–3000 masl. These results suggest that grafting can substantially affect the moisture content of tree tomato fruit, although variations may depend on the specific altitude and environmental conditions. These results highlight the importance of considering the relationship between grafting and moisture in tree tomato production. They may help guide agronomic practices to obtain fruit with desired moisture characteristics.

Regarding altitude, it was observed that the highest moisture levels were found in control plants at an altitude of 2260–2500 m in GOE and 2510–2750 m in GPE. At the same time, altitude significantly differed in both ecotypes’ fruit moisture in control and grafted plants. This effect suggests that photosynthesis of the two ecotypes is limited at extreme altitudes such as 2010–2250 masl and 2760–300 masl, resulting in lower water availability to the plant and, therefore, lower moisture content.

#### 3.2.7. Ash

The ash of tree tomato fruits of the ‘giant orange’ ecotype (GOE) ranged from 1.3 to 13.2%, while that of the fruits of the ‘giant purple’ ecotype (GPE) ranged from 0.9 to 3.4% (Table 4). The ash values obtained in this study were higher than those reported by other authors. For example, ash values of 0.8% were reported for the golden yellow variety and 0.9% for the purple-red variety purchased in the main markets of Tungurahua and Pichincha in Ecuador [10]. This difference may be due to the agronomic conditions of the crop and the supply of minerals during plant growth, as shown in Table 2 and Table 3. On the other hand, a literature review reported an ash range of 0.7 to 0.9% in the pulp of giant orange and giant purple tomatoes [5]. This difference may be because the results presented by other authors do not include skin and seed jelly, resulting in lower values.

Regarding grafting, it was observed that in GOE, tree tomato ash in control plants ranged from 1.5 to 13.2%, while in grafted plants it ranged from 1.3 to 3.2%. On the other hand, in GPE, ash in tree tomato ranged from 0.9 to 3.3% in control plants and from 1.6 to 3.4% in grafted plants. In most cases, the relationship between the control and graft groups significantly affected ash content, except at altitudes of 2260–2500 masl at GPE. These results may be relevant for producing and selecting tree tomatoes with desired ash profiles. They could provide helpful information for developing crop management and nutritional strategies.

Regarding altitude, it was observed that the highest ash values were found at altitudes of 2260–2500 masl in the GOE, while in the GPE, the highest values were found at altitudes of 2010–2250 masl. In turn, altitude showed a significant difference in fruit moisture in both ecotypes’ control and grafted groups.

#### 3.2.8. Maturity Indices

The maturity indices of tree tomato fruits of GOE ranged from 5.1 to 12.6, while that of GPE ranged from 7.5 to 18.1 (Table 4). The ability to achieve an adequate maturity index in tree tomatoes is crucial for their marketability and consumption. These results indicate that the fruits reached an acceptable maturity index according to INEN 1909, which establishes a minimum of 4.5 (°Brix/citric acid), and are in line with the observations of other authors [45].

Regarding grafting, it was observed that the maturity indices of tree tomato GOE ranged from 6.3 to 10.1 in control plants, while in grafted plants it ranged from 5.1 to 12.6. On the other hand, in GPE, the tree tomato maturity indices ranged from 8.9 to 13.6 for control plants and from 7.5 to 18.1 for grafted plants. In all cases, for both GOE and GPE, no significant effect of the ratio between the control and graft group on the maturity index was observed. These results are relevant, as the maturity index is an important indicator to determine the quality and maturity status of the fruit. 

Regarding altitude, it was observed that the highest values of the maturity indices were found in plants of the grafted group, at an altitude of 2010–2250 masl in GOE and 2260–2500 masl in GPE. At the same time, altitude showed a significant difference in the fruit maturity indices in both the control and grafted group in both ecotypes.

#### 3.2.9. Colour

Regarding fruit maturity, the colour of the samples was evaluated (Figure 1). The lightness (L*) of tree tomato fruits of the ‘giant orange’ ecotype (GOE) ranged from 40.2 to 63.9, while that of the fruits of the ‘giant purple’ ecotype (GPE) ranged from 37.2 to 53.7 (Figure 1). Chroma (C*_ab_) in GOE ranged from 27.8 to 54.1, while in GPE, it ranged from 27.0 to 32.2. In turn, the tone (h_ab_) in GOE ranged between 40.1 and 78.5, while in GPE between 25.6 and 71.9. Thus, the analysis of colour parameters is essential as it provides information on the appearance and visual quality of the fruit, considering that fruit quality is the sum of different parameters related to the photosynthetic process, which influence the acceptance of the fruit by the consumer [3,26,46]. The values obtained in this study are similar to those reported by Llerena et al. [43], who found an L* value of 51.8 and a h_ab_ value of 33.9 in tree tomatoes. The results obtained in this study on colour contribute to a better characterisation and understanding of the colourimetric aspect of tree tomatoes, which is relevant both for agricultural production and for the consumption and marketing of these fruits. 

Regarding grafting, it was observed that in GOE, the value of lightness (L*) ranged from 40.2 to 61.2 in the control group, and from 44.9 to 63.9 in the grafted group. For chroma (C*_ab_), values between 29.7 and 53.4 were observed in the control group and between 27.8 and 54.1 in the grafted group. For the tone parameter (h_ab_), values ranged from 40.1 to 78.0 in the control group and from 40.7 to 78.5 in the graft group. For GPE, L* values ranged from 37.2 to 49.8 in the control group and from 37.9 to 53.7 in the graft group. C*_ab_ values ranged from 27.2 to 29.5 in the control group and from 27.0 to 32.2 in the graft group. Finally, the h_ab_ parameter showed values between 25.6 and 71.9 in the control group and between 26.9 and 68.7 in the graft group. Furthermore, in most cases, colour parameters showed no statistical difference between control and grafted plants, except for L* in GPE at an altitude of 2510–2750 masl. 

Regarding altitude, the highest L* values were observed in the grafted group plants at 2010–2250 masl in GOE and 2510–2750 masl in GPE. C*_ab_ in GOE showed high values at 2010–2250 masl in control and grafted plants, while in GPE, high values were shown at 2510–2750 masl in grafted plants. Finally, h_ab_ in GOE showed high values at 2010–2250 and 2510–2750 masl in control plants, while in GPE at 2260–2500 masl in control and grafted plants. These data suggest that tree tomato fruits at lower altitudes showed brighter colours with higher intensity of red and yellow colour in both ecotypes.

### 3.3. Bioactive Compounds Quantification

Data on bioactive compounds such as vitamin C, organic acids, phenolics and carotenoids, as well as in vitro antioxidant activity, reported in the different parts of the fruit, such as peel, pulp, and seed jelly, are presented in Table 5 for the tree tomato ecotype ‘giant orange’ (GOE) and in Table 6 for the tree tomato ecotype ‘giant purple’ (GPE).

#### 3.3.1. Vitamin C Quantification

Total vitamin C as the sum of the individual concentrations of shell, pulp and seed jelly of tree tomato fruits of GOE ranged from 34.7 to 217.6 mg/100 g dry weight (DW) (Table 5), while vitamin C in fruits of GPE ranged from 56.7 to 175.0 mg/100 g DW (Table 6). It is important to note that the total vitamin C content was higher than that reported in other studies. Previous studies showed that the vitamin C content of tree tomatoes ranged from 30.0 to 45.0 mg/100 g in varieties ranging from yellow to orange-red, and from 19.7 a 57.8 mg/100 g in the yellow variety [9]. In addition, other authors have reported lower values, such as 17.0 mg/100 g fresh weight (FW) in the golden yellow variety and 16.0 mg/100 g FW in the purple red variety [10]. 

Regarding grafting, it was observed that the vitamin C content of tree tomato in GOE ranged from 34.7 to 217.6 mg/100 g DW in control plants and from 89.3 to 159.5 mg/100 g DW in grafted plants. On the other hand, in GPE, vitamin C in control plants ranged from 56.7 to 164.2 mg/100 g DW and in grafted plants from 80.5 to 175.0 mg/100 g DW. In all cases, for both GOE and GPE, a significant effect of the ratio between the control and grafted group on vitamin C content was observed. The results obtained in this study were lower than those reported by other authors, who found a vitamin C content of 209.8 mg/100 g FW in a segregating population of grafted plants on *Nicotiana glauca* grown at 2340 masl [11]. 

Regarding the fruit part, it was observed that the vitamin C content of tree tomato in GOE shell ranged from non-detectable limit (lnd) to 20.2 mg/100 g DW. GOE pulp ranged from 0.7 to 32.5 mg/100 g DW. GOE seed jelly ranged from 22.4 to 186.8 mg/100 g DW. GPE shell ranged from borderline undetectable to 22.5 mg/100 g DW. GPE pulp ranged from 0.8 to 48.6 mg/100 g DW. GPE seed jelly ranged from 29.3 to 133.9 mg/100 g DW. In turn, the highest levels of vitamin C were found in the seed jelly of the two tree tomato ecotypes, with approximately 90% more than in the GOE pulp, and 70% more than in the GPE pulp. It is important to mention that the vitamin C results in this study were similar to those reported by other authors [40], who found a value of 25 mg/100 g DW in the shell.

Regarding altitude, it was observed that the highest levels of vitamin C in GOE tomato were found in plants of the control group at an altitude of 2010–2250 masl and 2260–2500 masl. On the other hand, in GPE tomatoes, the highest concentrations of vitamin C were found in the fruits of plants grafted at 2510–2700 masl. At the same time, altitude showed a significant difference in the vitamin C content of the fruits of control and grafted plants in the two ecotypes. These findings align with those of Mphahlele et al. [47], who suggest that optimal vitamin C accumulation in fruit occurs during photosynthesis from sugars and is favoured under higher light-intensity growing conditions. In addition, altitudes above or below the recommended average can cause plant stress, which increases bioactive compound concentration. Orqueda et al. [48] showed that the pulp of yellow tree tomatoes grown in Argentina at altitudes below 500 masl had a higher concentration of ascorbic acid. These results suggest that environmental and altitude conditions influence the vitamin C content of tree tomatoes.

#### 3.3.2. Organic Acid Quantification

The total organic acid content, as the sum of citric, malic and tartaric acids in shell, pulp and seed jelly of tree tomato fruits of the ‘giant orange’ ecotype (GOE) ranged from 48.4 to 94.0 g/100 g DW (Table 5), while organic acid in fruits of the ‘giant purple’ ecotype (GPE) ranged from 42.0 to 115.8 g/100 g DW (Table 6).

The citric acid in GOE ranged from 4.7 g/100 to 20.0 g/100 g DW. The malic acid in GOE ranged from 0.1 to 1.4 g/100 g DW, while tartaric acid ranged from 3.3 to 27.7 g/100 g DW. In GPE, citric acid ranged from 3.4 to 18.8 g/100 g DW, malic acid from 0.1 to 1.4 g/100 g DW, and tartaric acid ranged from 2.4 to 27.6 g/100 g DW. 

The citric acid and malic acid concentrations in the control fruit reported in this study were higher than those reported by other authors for the golden yellow and purple-red varieties purchased in Ecuadorian and Spanish markets [14]. This difference could be attributed to the maturity of the fruit, since soluble solids, as an indicator of maturity, showed higher values in this study, leading to a higher accumulation of organic acids. In addition, it has been observed that organic acids accumulate during ripening, but are consumed after harvest due to fruit respiration [49]. 

Tartaric acid was predominant in both ecotypes, followed by citric acid. This characteristic is rare in plant species but has been observed in other fruits such as avocado, lychee, sweet cherry, blueberry, tamarind, and some citrus fruits. This peculiarity gives tree tomatoes unique characteristics. Although it is not fully understood how the accumulation of tartaric acid occurs, it is believed that one of the metabolic pathways is involved in ascorbic acid [50,51]. This characteristic may provide an antimicrobial effect in tree tomatoes, as citric acid has been shown to have antimicrobial properties [52]. 

Regarding grafting, it was observed that the total organic acid content of GOE ranged from 63.7 to 94.0 g/100 g DW in control plants and from 48.4 to 93.8 g/100 g DW in grafted plants. On the other hand, in GPE, organic acids ranged from 42.0 to 87.0 g/100 g DW in control plants and from 76.3 to 115.8 g/100 g DW in grafted plants. In all cases, for both GOE and GPE, a significant effect of the ratio between the control and grafted group on total organic acid content was observed. In addition, the control/graft ratio had a significant effect in some cases on the citric acid, malic acid and tartaric acid content in shell, pulp, and seed jelly. In contrast, the control/graft ratio significantly affected total organic acid content in all cases in both ecotypes. These results suggest that grafting can influence the composition of organic acids in tree tomatoes, which may affect their sensory characteristics and potentially their antimicrobial properties.

Regarding the fruit part, it was observed that the total organic acid content in GOE peel ranged from 8.8 to 30.4 g/100 g DW, while in GPE it ranged from 6.1 to 33.1 g/100 g DW. The total organic acid content in GOE pulp ranged from 13.5 to 38.6 g/100 g DW, while in GPE it ranged from 15.1 to 48.2 g/100 g DW. Furthermore, the total organic acid content in GOE seed jelly ranged from 20.9 to 40.0 g/100 g DW, whereas in GPE it ranged from 20.8 g/100 g DW to 41.1 g/100 g DW. It is also important to note that pulp and seed jelly had the highest concentrations of total organic acids in GOE.

Regarding altitude, it was observed that the highest values of total organic acids in GOE were found in plants of the control group at an altitude of 2760–3000 mass and in grafted plants at 2510–2750 masl, whereas in GPE, the high concentrations of total organic acids were found in fruits of grafted plants at 2760–3000 masl. At the same time, altitude showed a significant difference in both ecotypes’ fruit organic acid content in control and grafted plants. Furthermore, the highest concentrations of organic acids were observed at higher altitudes, which is consistent with the lower reported soluble solids (Table 4) and in agreement with the reports of other authors who indicate that organic compounds are produced from sugars during photosynthesis [47]; therefore, the lower the amount of soluble solids, the higher the total organic compounds.

#### 3.3.3. Phenolics Content Quantification

Total phenolics as the sum of individual phenols from shell, pulp and seed jelly of tree tomato fruits of the ‘giant orange’ ecotype (GOE) ranged from 1308.5 to 23,588.6 mg/100 g DW (Table 5), whereas, the total phenolic compounds of the fruits of the ‘giant purple’ ecotype (GPE) ranged from 3088.4 to 13,323.0 mg/100 g DW (Table 6). The GPE relative to the GOE showed a 62% higher phenol concentration. The results of this study contrast with a value of 1.4 mg GAE/g of total phenolic compounds in yellow tomatoes [9].

The profile of phenolic compounds showed that luteolin, ferulic acid, and caffeic acid were the most abundant phenolics in tree tomatoes. Among them, ferulic acid showed the highest concentration in both ecotypes. These high concentrations of ferulic acid could be related to the biosynthesis of phenolic compounds, suggesting that caffeic acid is previously formed and leads to the formation of ferulic acid, which contributes to a higher concentration of this compound [53]. As for luteolin, ferulic acid and caffeic acid in GOE ranged from the non-detectable limit to 993.3, 10,793 and 10,340 mg/100 g DW, respectively. In GPE, luteolin, ferulic acid and caffeic acid they were ranged from non-detectable limits to 593.7, 6017.5 and 4380.6 mg/100 g DW, respectively.

Regarding grafting, it was observed that the total phenolic content of tree tomato in GOE ranged from 1308.5 to 23,589.0 mg/100 g DW in control plants and from 5281.0 to 14,007.1 mg/100 g DW in grafted plants. On the other hand, the content of total phenolics in GPE in fruits of control plants ranged from 5415.5 to 12,009.1 mg/100 g DW and in fruits of grafted plants from 3088.4 to 13,323.0 mg/100 g DW. Regarding the relationship between control and grafting, significant differences in the concentration of phenolic compounds were observed in most cases, except for the GOE pulp, which showed no significant differences at an altitude of 2010–2250 masl. The high maturity in GPE indicates a higher formation of phenolic compounds, as suggested by other authors’ studies since sugars act as a substrate for anthocyanin synthesis and acids stimulate the synthesis potential, which is important for fruit colouration. Furthermore, in this study, grafting increased soluble solids in both ecotypes, which could be attributed to the stress caused by grafting on the plant, leading to a more significant accumulation of soluble solids and phenolic compounds [54].

Regarding the fruit part, it was observed that the total phenolic content in GOE peel ranged from 687.1 to 22,126 mg/100 g DW, while in GPE, it ranged from 338.2 to 10,991.8 mg/100 g DW. The total phenol content in GOE pulp ranged from 385.3 to 1720.9 mg/100 g DW, whereas in GPE, it ranged from borderline undetectable to 3223.2 mg/100 g DW. In addition, the total phenolic content in GOE seed jelly showed 266.9 mg/100 g DW in control plants at 2760–300 masl, while GPE showed a non-detectable limit in all cases. The results obtained in the control of this study showed higher concentrations than those reported by other authors for phenolic compounds in the pulp (5.1 to 16.6 mg GAE/g) of a segregating population in Ecuador [11]. However, the results of this study are in line with other studies that reported values of 387.0, 78.0 and 94.0 mg GAE/100 g in the shell, pulp and seed jelly for the golden-yellow variety and values of 620.0, 113.0 and 152.0 mg GAE/100 g in the peel, pulp and seed jelly for the purple-red variety, respectively [10]. In turn, the highest concentrations of phenolic compounds were observed in the shell in most cases in GOE, while in GPE, the highest concentration was found in both the shell and the seed jelly.

Regarding altitude, it was observed that the highest values of total phenolics were found in plants of the grafted group at an altitude of 2260–2500 m in the two ecotypes. At the same time, altitude showed a significant difference in the content of total phenolics in the fruit of plants of the control group and plants of the grafted group in the two ecotypes.

#### 3.3.4. Carotenoids Content Quantification

Total carotenoids, as the sum of individual compounds, in shell, pulp, and seed jelly of tree tomato fruits of the ‘giant orange’ ecotype (GOE) ranged from 44.2 to 390.7 mg/100 g DW (Table 5), whereas, the total phenolic compounds of the fruits of the ‘giant purple’ ecotype (GPE) ranged from 67.4 to 208.7 mg/100 g DW (Table 6). 

The carotenoid profile showed that lutein, β-cryptoxanthin, and β-carotene were the most abundant carotenoids in tree tomatoes. As for lutein in GOE, it ranged from 5.2 to 113.6 mg/100 g DW, β-cryptoxanthin from 0.5 to 19. 5 mg/100 g DW and β-carotene ranged from 0.4 to 43.1 mg/100 g DW. Lutein in GPE ranged from 3.5 to 74.6 mg/100 g DW, β-cryptoxanthin from 0.5 to 13. 4 mg/100 g DW and β-carotene ranged from 0.5 to 162.6 mg/100 g DW. 

Regarding grafting, it was observed that the total carotenoid content of tree tomato in GOE ranged from 73.5 mg/100 g DW to 390.7 mg/100 g DW in control plants and from 44.2 to 112.0 mg/100 g DW in grafted plants. On the other hand, in GPE, it ranged from 162.6 to 208.7 mg/100 g DW in control plants and from 67.4 to 82.3 mg/100 g DW in grafted plants. Significant differences in carotenoid concentration were observed in all cases between control and grafted plants. In addition, grafting caused a decrease in carotenoids in most cases in both ecotypes, except at a height of 2510–2750 masl in GOE. GOE treatment affected β-cryptoxanthin concentration in all cases, while lutein and β-carotene were affected in most cases in all three study fractions.

Regarding the fruit part, the total carotenoid content in GOE peel ranged from 7.2 to 122.7 mg/100 g DW, while in GPE, it ranged from 8.2 to 79.3 mg/100 g DW. Total carotenoids in GOE pulp ranged from 14.3 to 147.0 mg/100 g DW, whereas in GPE, it ranged from 17.3 to 73.2 mg/100 g DW. Furthermore, the total carotenoid content in GOE seed jelly ranged from 16.1 to 54.4 mg/100 g DW, while in GPE, it ranged from 24.9 to 96.3 mg/100 g DW. These carotenoid levels were higher than those found in the pulp (0.93 g β-carotene/100 g DW), shell (1.37 g β-carotene/100 g DW), and seed jelly (0.53 g β-carotene/100 g DW) of yellow tomatoes grown in Argentina [48]. They are also related to β-carotene concentrations in the pulp (0.4, 0.8 and 0.9 mg/100 g of fresh weight (FW) of Laird’s, Amber and Mulligan variety, respectively) and shell (0.4 to 0.5 mg/100 g FW) of tomatoes grown in New Zealand [6], and in the pulp (50.4 to 460.7 µg β-carotene/g) of segregants grown in Ecuador [11].In addition, higher concentrations of lutein were observed in all three study fractions (shell, pulp, seed jelly) compared to the other two carotenoids. In most cases, β-cryptoxanthin showed high pulp and seed jelly concentrations, while β-carotene was more abundant in seed jelly in the GOE. These results are consistent with those reported by Mertz [55].

Regarding altitude, it was observed that the highest values of total carotenoids were found in plants of the control group at an altitude of 2760–3000 m in GOE and 2260–2500 m in GPE. In turn, altitude showed a significant difference in the total carotenoid content of the fruit in plants of the control group and plants of the grafted group in the two ecotypes.

### 3.4. Antioxidant Activity Determination

#### 3.4.1. Antioxidant Activity by ABTS Assay

The antioxidant activity measured by ABTS assay of tree tomatoes of ‘giant orange’ ecotype (GOE) ranged from 4.4 to 119.7 µmol Trolox equivalent (TE)/g DW (Table 5), while that of fruits of ‘giant purple’ ecotype (GPE) ranged from 3.4 to 137.9 µmol TE/g DW (Table 6). 

Regarding grafting, it was observed that the antioxidant activity of tree tomato in GOE, control plants in peel, pulp and seed jelly ranged from 39.8 to 117. 4 µmol TE/g DW, 7.0 to 31.9 µmol TE/g DW, 24.2 to 79.1 µmol TE/g DW, respectively. In turn, in plants grafted on GOE shell, pulp and seed jelly, it ranged from 92.7 to 109.5 µmol TE/g DW, 4.4 to 16.7 µmol TE/g DW, 14.5 to 119.7 µmol TE/g DW. On the other hand, the antioxidant activity of tree tomato in GPE, in control plants in peel, pulp and seed jelly ranged from 70.8 to 137. 9 µmol TE/g DW, 3.4 to 18.9 µmol TE/g DW, 105.5 to 119.8 µmol TE/g DW, respectively. In turn, in shell-grown plants, pulp and seed jelly of GPE ranged from 10.7 to 137.1 µmol TE/g DW, 4.1 to 32.8 µmol TE/g DW, 91.5 to 93.2 µmol TE/g DW, respectively. In all cases, for both GOE and GPE, a significant effect of the ratio between the control and the graft group on the antioxidant activity was observed, except for the peel of GPE at 2260–2500 masl.

Regarding the fruit part, it was observed that the antioxidant activity of tree tomato in GOE, in the peel, ranged from 39.8 to 117.4 µmol TE/g DW, while in GPE, it ranged from 10.7 to 137.9 µmol TE/g DW. The antioxidant activity in GOE pulp ranged from 4.4 to 31.9 µmol TE/g DW, whereas in GPE, it ranged from 3.4 to 32.8 µmol TE/g DW. Furthermore, the antioxidant activity in GOE seed jelly ranged from 14.5 to 119.7 µmol TE/g DW, whereas, in GPE, it ranged from 91.5 to 119.8 µmol TE/g DW. In the GOE, the control shell reached the highest antioxidant capacity values at 2510–2750 masl altitude. These values are related to those found by Viera et al. [11] in segregants grown at 2340 masl (49.5 to 312.3 µmol TE/g). 

Regarding altitude, it was observed that the highest values of antioxidant activity occurred at an altitude of 2010–2250 masl in plants of the grafted group in GOE and fruits of grafted and control plants in GPE. At the same time, altitude showed a significant difference in fruit antioxidant activity in the two ecotypes of control and grafted group plants.

#### 3.4.2. Antioxidant Activity by DPPH Assay

Antioxidant activity, as measured by the DPPH assay of tree tomatoes of ‘giant orange’ ecotype (GOE) ranged from 4.9 to 31.4 µmol ascorbic acid equivalent (AAE)/g DW (Table 5), while that of fruits of ‘giant purple’ ecotype (GPE) ranged from 7.3 to 47.1 µmol AAE/g DW (Table 6). The antioxidant activity in both ecotypes was mainly related to the concentration of phenolic compounds, as suggested by several authors [56,57]. In addition, the high antioxidant activity of GOE was also associated with high concentrations of organic acids and carotenoids found in this study.

Regarding grafting, it was observed that the antioxidant activity of tree tomato in GOE, control plants in shell, pulp and seed jelly ranged from 4.9 to 31.4 µmol AAE/g DW, 10.3 to 20.5 µmol AAE/g DW, 9.0 to 13.8 µmol AAE/g DW, respectively. In turn, in plants grafted on shell, pulp and seed jelly in GOE, the values ranged from 23.7 to 26.3 µmol AAE/g DW, 7.6 to 18.6 µmol AAE/g DW, 9.9 to 21.2 µmol AAE/g DW. In the case of tree tomato antioxidant activity in GPE, in control plants in shell, pulp and seed jelly ranged from 19.4 to 22.9 µmol AAE/g DW, 7.3 to 14.0 µmol AAE/g DW, 19.5 to 47.1 µmol AAE/g DW. In turn, in plants grafted on shell, pulp and seed jelly in GPE, the values ranged from 7.6 to 21.9 µmol AAE/g DW, 8.2 to 15.6 µmol AAE/g DW, 18.5 to 45.8 µmol AAE/g DW. In all cases, except for pulp at 2510–2750 masl, the graft-control ratio showed a significant effect on GOE, while only at 2510–2750 masl for GPE was a significant effect of the ratio between the control and graft group on antioxidant activity.

Regarding the fruit part, it was observed that the antioxidant activity of tree tomato in GOE, in the shell, ranged from 4.9 to 31.4 µmol AAE/g DW, while in GPE, it ranged from 7.6 to 22.9 µmol AAE/g DW. Antioxidant activity in GOE pulp ranged from 7.6 to 20.5 µmol AAE/g DW, while in GPE, it ranged from 7.3 to 15.6 µmol AAE/g DW. In addition, antioxidant activity in GOE seed jelly ranged from 9.0 to 21.2 µmol AAE/g DW, whereas in GPE it ranged from 18.5 to 47.1 µmol AAE/g DW. In GOE, the antioxidant activity was mainly higher in the shell, whereas in GPE, it was higher in the seed jelly. These results are in agreement with the findings of Vasco et al. [10], who determined the antioxidant capacity using the DPPH method and reported values of 22.0, 2.3, and 3.8 µmol/g fresh weight (FW) for shell, pulp, and seed jelly, respectively, for the golden-yellow cultivar. For the purple-red cultivar, the values were 40.0, 3.0 and 9.3 µmol Trolox/g FW for shell, pulp, and seed jelly, respectively, with shell showing the highest and pulp the lowest values, as in this study. 

Regarding altitude, it was observed that the highest values of antioxidant activity were found in plants of the control group, at an altitude of 2760–300 masl in GOE and 2260–2500 masl in GPE. At the same time, altitude showed a significant difference in the antioxidant activity of the fruit in plants of the control group and plants of the grafted group in the two ecotypes.

### 3.5. Correlation, Multifactorial and Principal Component Analysis

**Figure 2:**
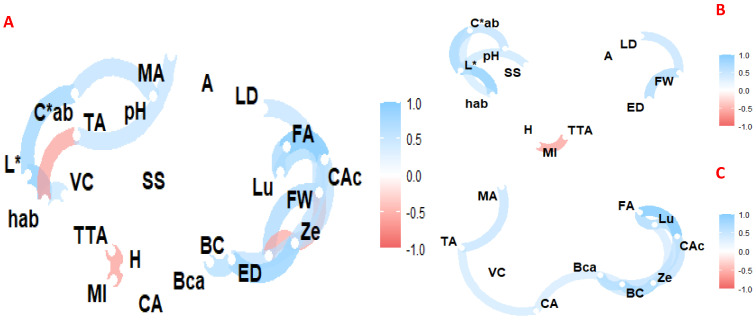
Pearson correlation of all studied variables (**A**), commercial quality (**B**) and bioactive compounds (**C**). Note: ED: equatorial diameter; LD: longitudinal diameter; FW: fruit weight; H: humidity; A: ash; SS: soluble solids; TTA: total titratable acidity; MI: maturity index; VC: vitamin C; CA: citric acid; MA: malic acid; TA: tartaric acid; Lu: luteolin; FA: ferulic acid; CAc: caffeic acid; Ze: Zeaxanthin; BC: β–cryptoxanthin; BCa: β–carotene.

**Figure 3:**
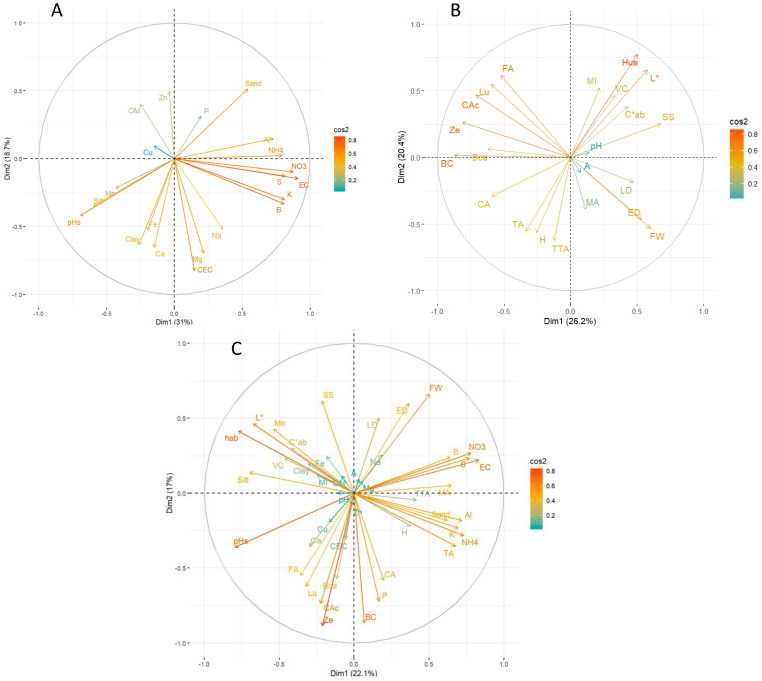
Exploratory multivariate analysis using PCA ((**A**): soil; (**B**): commercial quality and bioactive compounds, and (**C**): all results). EC: electric conductivity (mmhos/cm); OM: organic material; CEC: cation exchange capacity; ED: equatorial diameter; LD: longitudinal diameter; FW: fruit weight; H: humidity; A: ash; SS: soluble solids; TTA: total titratable acidity; MI: maturity index; VC: vitamin C; CA: citric acid; MA: malic acid; TA: tartaric acid; Lu: luteolin; FA: ferulic acid; CAc: caffeic acid; Ze: zeaxanthin; BC: β–cryptoxanthin; BCa: β–carotene.

Pearson linear correlation coefficients are shown in Figure 2. The analysis of variance (multifactorial ANOVA) controlling for altitude, ecotype, control and grafting, and part of the fruit is shown in Table 7, and the exploratory multivariate analysis using principal components analysis (PCA) is shown in Figure 3

Correlation analysis of commercial quality (Figure 2) showed, for example, a positive relationship of L* with C_ab_*, h_ab_, pH and soluble solids, weight with fruit size, as reported in numerous studies. In contrast, a negative relationship was reported between the maturity index with moisture and total titratable acidity. In the case of bioactive compounds, a positive relationship was found between tartaric acid with malic acid and vitamin C, β-carotene with citric acid and β-cryptoxanthin, caffeic acid with zeaxanthin and luteolin. In addition, pH was correlated with malic and tartaric acid, caffeic acid with weight, zeaxanthin and ferulic acid.

The multifactorial analysis showed that commercial quality, equatorial diameter, pH, and maturity index were mainly influenced by altitude, ecotype, and control graft variables (Table 7). Altitude mainly influenced the longitudinal diameter, h_ab_ and C_ab_*, while the control graft influenced weight. Ash and titratable acidity were influenced by altitude and ecotype, while L* and soluble solids were influenced by altitude and control graft. As for bioactive compounds, vitamin C, ferulic acid and beta-cryptoxanthin were mainly influenced by the fruit part. The height and fruit part influenced citric acid, while all three variables influenced malic acid. Tartaric acid and caffeic acid were influenced by altitude, control graft and fruit part, while the control graft and fruit part influenced lutein and β-carotene.

Principal component analysis (PCA) based on the interactions between soil characteristics (Figure 3A), commercial fruit quality and bioactive compound (Figure 3B) and the interaction of all analysed parameters (Figure 3C) showed a total variance for each case of 49.7%, 46.6% and 39.1%, respectively. The PCA revealed that zinc had a positive contribution. In contrast, cation exchange capacity negatively contributes to soil component 1 (Figure 3A). On the other hand, ammonium, nitrate, electrical conductivity, potassium, and boron had a positive contribution, while pH had a negative contribution to component 2. Regarding fruit quality (Figure 3B), colour (h_ab_ and L*) positively contributed to component 1, while moisture and titratable acidity had a negative contribution. Lutein, β–cryptoxanthin and β-carotene also had a negative contribution to component 2. An overall analysis of all parameters evaluated showed that soluble solids, equatorial diameter, and fruit weight had a positive contribution, while carotenoids and phenolic compounds had a negative contribution to component 1 (Figure 3C). In addition, soil parameters had a positive contribution, and both soil pH and fruit colour (h_ab_ and L*) had a negative contribution to component 2.

Correlation analysis in this study revealed several significant associations. Soil parameters showed a higher correlation with bioactive compounds than with the marketable quality of the fruit (Figure 3A,C). For example, soil pH was positively correlated with soil organic matter and manganese, and with ash, colour, soluble solids, citric acid and β-cryptoxanthin in the fruit. In addition, soil electrical conductivity was correlated with soil calcium, iron, and manganese, as well as with ash, citric acid, soluble solids, luteolin and β-cryptoxanthin in the fruit. On the other hand, soil phosphorus was correlated with soil calcium, cation exchange capacity and soil iron, as well as with soluble solids, vitamin C, luteolin and β-carotene in the fruit. Similarly, a correlation was found between soil nitrate concentration and soil phosphorus, calcium, magnesium, cation exchange capacity, iron, and zinc, as well as with ash, soluble solids, maturity index, ferulic acid and caffeic acid in the fruit. Regarding colour parameters, a correlation was observed between L* and the presence of luteolin, caffeic acid and lutein, while C_ab_* correlated with citric acid, ferulic acid, caffeic acid and lutein. In addition, a correlation was found between the longitudinal diameter of the fruit and several parameters, including moisture, C_ab_*, soluble solids, pH, total titratable acidity, vitamin C, citric acid, malic acid and β-cryptoxanthin.

These correlations highlight the close relationship between soil parameters and bioactive compounds present in the fruit, which is relevant for understanding and optimising the nutritional quality of crops. Several studies have documented a significant relationship between soil electrical conductivity and root nutrient uptake. A high value of electrical conductivity indicates that the roots must make a greater effort to obtain the necessary nutrients, which can cause additional stress in the plant and affect the biosynthesis of secondary metabolites such as phenolic compounds [57]. In addition, it has been observed that high nitrogen levels in the soil can induce stress in the plant, resulting in higher concentrations of secondary metabolites, as mentioned above. These findings are consistent with previous research that has found direct relationships between different soil parameters. For example, direct relationships have been reported between pH and manganese [36], pH and organic matter [38], electrical conductivity and calcium [23], and phosphorus, pH and calcium [58]. In addition, iron, calcium and soil pH are known to influence iron availability to plants [59]. Positive correlations have also been reported between fruit colour and the presence of compounds such as carotenoids and flavonoids, as noted by other researchers in previous studies [3,31,60,61].

## 4. Conclusions

The tree tomato is a fruit with a high concentration of metabolites and could therefore be considered a superfood. However, its cultivation is threatened by pests, which makes the use of grafting essential. Despite its potential, attention has mainly focused on the pulp of this fruits, leaving aside other parts that could be used in various industrial applications. Therefore, the aim of this study was to evaluate the bioactive compounds of the fruit (shell, pulp, and seed jelly) of two ecotypes of tree tomatoes (‘giant orange’ and ‘giant purple’), considering control and grafted plants and their development at different growing altitudes (2010–2250, 2260–2500, 2510–2750 and 2760–3000 masl). There were remarkable similarities between GOE and GPE regarding weight, size, and soluble solids content. However, GOE showed higher contents of total vitamin C, total phenolic compounds and total carotenoids compared to GPE. In addition, the phenolic profiles in GOE and GPE reported concentrations of luteolin, ferulic acid and caffeic acid, and the carotenoid profiles reported concentrations of lutein, β-cryptoxanthin and β-carotene. About grafting, grafted plants of both ecotypes showed higher values for weight, size, soluble solids, total vitamin C and antioxidant activity in seed jelly. In terms of fruit part, GOE control plants showed the highest total ascorbic acid content in seed jelly, while total phenolic compounds and carotenoids were more abundant in the shell. In GPE, the highest antioxidant activity was found in the shell of control plants. About altitude, GOE showed better marketable quality at 2260–2500 masl, higher total vitamin C at 2010–2250 masl and high levels of organic acids, total phenolic compounds, total carotenoids and antioxidant activity in shell and pulp at 2760–3000 masl. Finally, multifactorial analysis showed that altitude and fruit part had a strong influence on fruit quality. Furthermore, principal component analysis showed that component 1 was positively influenced by soluble solids, equatorial diameter, and weight, and negatively by carotenoids and phenolic compounds, while component 2 was positively and negatively influenced by soil parameters. These results will contribute to the knowledge of the fruit quality and phytochemical components of the tree tomato ecotypes ‘giant orange’ and ‘giant purple’, promoting the consumption of this exotic fruit due to the content of bioactive compounds and their nutraceutical value, favouring the health of consumers. 

## Figures and Tables

**Table 1:** Location of experimental plots of the ‘giant orange’ (GOE) and ‘giant purple’ (GPE) tree tomato ecotypes.

Altitude Range (Masl)	Sampling Site			T (°C)	Altitude (masl)	Soil Texture
Control ‘giant orange’ ecotype						
2010–2250	Juive Chico	S 1° 24′49″	W 78° 28′22″	17	2141	Loamy sand
2260–2500	Runtun	S 1° 24′48″	W 78° 24′56″	15	2385	Loamy sand
2510–2750	Pisque	S 1° 12′15″	W 78° 32′10″	12	2700	Loamy sand
2760–3000	Ciudad Nueva	S 1° 10′34″	W 78° 33′26″	12	2756	Loamy sand
Grafting ‘giant orange’ ecotype						
2010–2250	Juive Chico	S 1° 24′49″	W 78° 28′22″	17	2141	Loamy sand
2260–2500	Runtun	S 1° 24′37″	W 78° 31′33″	15	2347	Loamy sand
2510–2750	Capulicito	S 1° 21′39″	W 78° 38′22″	12	2616	Loamy sand
2760–3000	Pillaro centro	S 1° 11′0.9″	W 78° 32′29″	12	2780	Loamy sand
Control ‘giant purple’ ecotype						
2260–2500	Bellavista	S 1° 16′59″	W 78° 39′43″	15	2500	Loamy sand
2510–2750	Huasapamba	S 1° 21′39″	W 78° 31′38″	15	2595	Loamy sand
2760–3000	Pichimbana	S 1° 21′57″	W 78° 32′35″	12	2772	Loamy sand
Grafting ‘giant purple’ ecotype						
2260–2500	Chiquicha bajo	S 1° 15′6″	W 78° 32′13″	15	2500	Loamy sand
2510–2750	Bellavista	S 1° 17′37″	W 78° 39′21″	15	2546	Loamy sand
2760–3000	Olmedo	S 1° 20′59″	W 78° 32′42″	12	2762	Loamy sand

**Table 2:** Average values of the soil analysis of the ‘giant orange’ tree tomato ecotype (GOE) at different altitudes.

Altitude Range (Masl)	2010–2250			2260–2500			2510–2750			2760–3000			A_AC_	A_AG_
	Control	Grafting	A_CG_	Control	Grafting	A_CG_	Control	Grafting	A_CG_	Control	Grafting	A_CG_		
Sand (%)	53.3 ± 1.0	53.5 ± 1.3	ns	57.8 ± 1.3	56.0 ± 0.8	ns	44.0 ± 0.8	62.0 ± 0.8	***	53.8 ± 0.5	52.3 ± 0.5	ns	***	***
Clay (%)	14.0 ± 0.8	11.3 ± 1.0	**	16.0 ± 0.8	18.5 ± 1.3	ns	28.8 ± 1.0	15.5 ± 0.6	***	14.8 ± 1.0	18.5 ± 0.6	**	***	***
Silt (%)	32.0 ± 1.4	34.3 ± 1.7	ns	26.8 ± 1.0	25.5 ± 0.6	ns	28.3 ± 1.0	22.3 ± 0.5	*	31.5 ± 1.3	29.0 ± 0.8	ns	***	***
pHs	6.2 ± 0.1	6.1 ± 0.1	ns	5.5 ± 0.1	**5.3** ± 0.1	ns	6.4 ± 0.1	**5.3** ± 0.1	*	**6.9** ± 0.1	6.2 ± 0.1	ns	***	***
EC	**0.9** ± 0.1	1.8 ± 0.1	***	3.2 ± 0.1	5.3 ± 0.1	*	2.2 ± 0.1	**9.1** ± 0.1	***	1.4 ± 0.1	6.4 ± 0.1	***	***	***
OM (%)	3.3 ± 0.1	2.2 ± 0.1	***	**5.8** ± 0.1	4.3 ± 0.1	ns	2.4 ± 0.1	1.3 ± 0.1	***	2.4 ± 0.0	2.8 ± 0.1	ns	***	***
CEC (meq/100 mL)	12.9 ± 0.0	11.4 ± 0.1	ns	12.1 ± 0.0	17.9 ± 0.1	ns	24.6 ± 0.0	22.6 ± 0.0	ns	23.6 ± 0.0	22.9 ± 0.0	ns	**	***
(NH_4_)^+^ (ppm	51.9 ± 0.1	33.2 ± 0.1	***	50.4 ± 0.1	53.5 ± 0.1	ns	**25.4** ± 0.1	**116.1** ± 0.1	***	64.5 ± 0.1	42.6 ± 0.1	***	***	***
(NO_3_)^−^ (ppm)	**56.1** ± 0.1	135.8 ± 0.1	***	184.6 ± 0.1	365.7 ± 0.1	***	113.7 ± 0.1	**463.4** ± 0.2	***	72.3 ± 0.0	215.8 ± 0.1	***	***	***
P^3−^ (ppm)	342.5 ± 63.3	224.0 ± 41.3	ns	164.5 ± 30.4	332.5 ± 61.4	*	**55.4** ± 10.3	401.5 ± 76.7	**	**625.0** ± 115.5	127.0 ± 23.4	**	***	***
K^+^(meq/100 mL)	0.7 ± 0.1	**0.5** ± 0.1	ns	0.9 ± 0.2	1.6 ± 0.3	ns	1.4 ± 0.3	**4.2** ± 0.9	*	2.3 ± 0.5	2.8 ± 0.6	ns	**	***
Ca^2+^(meq/100 mL)	8.9 ± 1.8	8.6 ± 1.8	ns	**7.0** ± 1.5	12.8 ± 2.7	*	**17.6** ± 3.7	10.4 ± 2.2	ns	16.9 ± 3.6	14.9 ± 3.1	ns	**	ns
Mg^2+^(meq/100 mL)	2.8 ± 0.6	**1.9** ± 0.3	ns	3.5 ± 0.7	2.9 ± 0.6	ns	5.1 ± 1.0	**6.9** ± 1.4	ns	3.8 ± 0.7	4.5 ± 0.9	ns	*	***
Mn^2+^ (ppm)	**9.8** ± 3.0	41.0 ± 1.3	***	31.5 ± 9.8	35.5 ± 11.1	ns	24.0 ± 7.5	**92.4** ± 2.8	***	10.1 ± 3.1	11.9 ± 3.7	ns	*	***
Fe^2+^ and Fe^3+^ (ppm)	131.9 ± 39.6	67.6 ± 20.3	ns	168.1 ± 50.5	169.2 ± 50.7	ns	1600.0 ± 138.7	152.5 ± 45.8	***	80.1 ± 23.8	33.2 ± 9.9	ns	***	***
Na^+^(meq/100 mL)	**0.1** ± 0.0	**0.1** ± 0.0	ns	**0.1** ± 0.0	0.2 ± 0.0	ns	**0.1** ± 0.0	0.3 ± 0.0	*	**0.1** ± 0.0	0.3 ± 0.0	*	ns	***
Al^3+^(meq/100 mL)	0.4 ± 0.0	0.4 ± 0.0	ns	0.5 ± 0.0	0.5 ± 0.0	ns	**0.3** ± 0.0	**0.7** ± 0.0	**	0.5 ± 0.0	0.4 ± 0.0	ns	ns	***
Cu^2+^ (ppm)	6.6 ± 1.5	7.2 ± 1.7	ns	6.3 ± 1.5	**11.1** ± 2.6	ns	5.1 ± 1.2	6.2 ± 1.4	ns	9.4 ± 1.9	3.5 ± 0.8	*	ns	**
Zn^2+^ (ppm)	26.6 ± 11.7	18.5 ± 8.1	ns	14.8 ± 6.5	**33.4** ± 14.7	ns	**2.4** ± 1.1	11.0 ± 4.8	ns	13.5 ± 5.9	4.2 ± 1.8	ns	*	*
B (ppm)	**1.8** ± 0.0	2.3 ± 0.0	***	3.2 ± 0.0	5.8 ± 0.1	*	3.5 ± 0.2	**8.4** ± 0.0	***	2.3 ± 0.0	4.3 ± 0.0	***	***	***
(SO_4_)^2−^ (ppm)	**8.1** ± 0.1	9.9 ± 0.0	***	47.1 ± 0.1	44.2 ± 0.1	ns	24.3 ± 0.1	**79.8** ± 0.0	***	14.3 ± 0.1	15.8 ± 0.1	ns	***	***

The range is shown in bold. EC: electric conductivity (mmhos/cm); OM: organic material; CEC: cation exchange capacity. Significance of differences between the control and graft (A_CG_); altitude in control (A_AC_); altitude in the graft (A_AG_) is given: ns, not significant, * *p* < 0.1, ** *p* < 0.01, *** *p* < 0.001.

**Table 3:** Average values of the soil analysis of the ‘giant purple’ tree tomato ecotype (GPE) at different altitudes.

Altitude Range (Masl)	2260–2500			2510–2750			2760–3000			A_AC_	A_AG_
	Control	Grafting	A_CG_	Control	Grafting	A_CG_	Control	Grafting	A_CG_		
Sand (%)	50.8 ± 1.0	61.5 ± 1.3	***	70.8 ± 1.0	44.0 ± 0.8	***	77.0 ± 1.4	76.8 ± 1.5	ns	***	***
Clay (%)	16.0 ± 0.8	12.0 ± 0.8	***	10.0 ± 0.8	20.5 ± 1.3	***	9.5 ± 1.3	8.8 ± 1.0	ns	***	***
Silt (%)	34.5 ± 1.3	27.5 ± 1.3	**	18.3 ± 1.5	35.8 ± 0.5	***	14.3 ± 0.5	13.8 ± 1.0	ns	***	***
pHs	6.5 ± 0.1	6.2 ± 0.1	ns	6.2 ± 0.1	6.4 ± 0.2	ns	5.5 ± 0.1	5.7 ± 0.1	ns	***	***
EC	2.7 ± 0.1	4.6 ± 0.1	***	1.9 ± 0.1	3.1 ± 0.1	***	6.0 ± 0.1	5.7 ± 0.1	ns	***	***
OM (%)	2.1 ± 0.1	1.7 ± 0.1	*	2.3 ± 0.1	1.7 ± 0.1	***	1.8 ± 0.1	**1.0** ± 0.0	***	**	***
CEC (meq/100 mL)	17.9 ± 0.0	22.9 ± 0.0	***	17.9 ± 0.0	22.2 ± 0.0	***	15.6 ± 0.0	14.4 ± 0.0	ns	***	***
(NH_4_)^+^ (ppm	50.4 ± 0.1	70.6 ± 0.0	***	72.4 ± 0.2	54.9 ± 0.1	***	52.0 ± 0.1	62.4 ± 0.5	ns	***	***
(NO_3_)^−^ (ppm)	146.4 ± 0.0	291.3 ± 0.2	***	83.4 ± 0.6	177.7 ± 0.2	***	238.5 ± 0.6	246.2 ± 0.2	ns	***	***
P^3−^ (ppm)	100.0 ± 18.5	339.0 ± 62.6	**	428.0 ± 79.1	109.5 ± 20.2	**	169.0 ± 31.2	187.0 ± 34.5	ns	***	***
K^+^ (meq/100 mL)	1.3 ± 0.2	2.4 ± 0.5	*	1.5 ± 0.3	1.5 ± 0.3	ns	2.3 ± 0.5	2.9 ± 0.6	ns	*	**
Ca^2+^ (meq/100 mL)	11.1 ± 2.3	15.2 ± 3.1	ns	11.1 ± 2.2	13.9 ± 2.9	ns	8.7 ± 1.8	8.1 ± 1.7	ns	ns	*
Mg^2+^ (meq/100 mL)	4.8 ± 0.9	4.6 ± 0.9	ns	4.8 ± 0.9	6.1 ± 1.2	ns	3.9 ± 0.8	2.8 ± 0.6	ns	ns	**
Mn^2+^ (ppm)	24.0 ± 1.3	6.5 ± 2.1	***	19.0 ± 5.9	25.0 ± 1.3	***	26.4 ± 8.2	35.2 ± 11.0	ns	***	***
Fe^2+^ and Fe ^3+^ (ppm)	**22.6** ± 6.8	101.2 ± 30.4	*	154.7 ± 46.4	**2500.0** ± 1.3	***	87.5 ± 26.3	72.3 ± 0.0	ns	**	***
Na^+^ (meq/100 mL)	**0.4** ± 0.0	0.3 ± 0.0	ns	**0.1** ± 0.0	0.3 ± 0.0	ns	**0.4** ± 0.0	0.2 ± 0.0	***	***	***
Al^3+^ (meq/100 mL)	0.3 ± 0.0	0.4 ± 0.0	ns	0.5 ± 0.0	0.4 ± 0.0	ns	0.4 ± 0.0	0.5 ± 0.0	ns	***	***
Cu^2+^ (ppm)	4.8 ± 1.1	6.6 ± 1.5	ns	7.1 ± 1.6	7.6 ± 4.4	ns	**3.6** ± 0.8	4.7 ± 1.1	ns	*	ns
Zn^2+^ (ppm)	6.8 ± 3.0	21.8 ± 9.6	ns	18.6 ± 8.2	4.5 ± 2.0	ns	5.0 ± 2.2	7.6 ± 3.3	ns	*	*
B (ppm)	4.8 ± 0.5	4.6 ± 0.0	ns	2.5 ± 0.0	3.1 ± 0.0	ns	3.5 ± 0.0	3.7 ± 0.0	ns	***	***
(SO_4_)^2−^ (ppm)	38.5 ± 0.1	30.0 ± 0.2	***	16.5 ± 0.1	30.5 ± 0.1	***	51.2 ± 0.1	28.7 ± 0.1	***	***	***

The range is shown in bold. EC: electric conductivity (mmhos/cm); OM: organic material; CEC: cation exchange capacity. Significance of differences between the control and graft (A_CG_); altitude in control (A_AC_); altitude in the graft (A_AG_) is given: ns, not significant, * *p* < 0.1, ** *p* < 0.01, *** *p* < 0.001.

**Table 4:** Average values of the commercial quality parameters of the ‘giant orange’ (GOE) and ‘giant purple’ (GPE) tree tomato ecotypes grown at different altitudes.

Altitude Range (Masl)	2010–2250			2260–2500			2510–2750			2760–3000			A_AC_	A_AG_
	Control	Grafting	A_CG_	Control	Grafting	A_CG_	Control	Grafting	A_CG_	Control	Grafting	A_CG_		
‘Giant orange’ ecotype (GOE)														
Fruit weight (g)	131.4 ± 19.6	131.8 ± 15.2	ns	**145.7** ± 19.6	**127.3** ± 24.4	ns	107.9 ± 21.3	**160.4** ± 29.3	***	**75.9** ± 20.6	154.4 ± 17.0	***	***	***
Pulp weight (g)	71.6 ± 11.7	76.2 ± 9.0	ns	**93.7** ± 14.4	**51.9** ± 16.4	***	63.9 ± 12.1	**93.6** ± 24.1	***	**49.5** ± 16.3	83.6 ± 11.0	***	***	***
Seed jelly weight (g)	**39.2** ± 6.9	**36.2** ± 10.5	ns	34.1 ± 8.2	**57.4** ± 19.8	***	28.2 ± 6.6	39.9 ± 8.9	***	**17.4** ± 5.4	51.3 ± 10.5	***	***	***
ED (cm)	5.8 ± 0.4	**5.8** ± 0.3	ns	**6.2** ± 0.4	**6.9** ± 0.7	**	5.5 ± 0.5	6.2 ± 0.4	***	**4.7** ± 0.5	6.1 ± 0.2	***	***	***
LD (cm)	**7.3** ± 0.4	7.3 ± 0.4	ns	**7.3** ± 0.7	**5.8** ± 0.8	***	6.9 ± 0.6	7.7 ± 0.6	**	**6.1** ± 0.7	**8.1** ± 0.7	***	***	***
pH	**5.8** ± 0.4	**6.0** ± 0.0	*	4.1 ± 0.1	3.9 ± 0.4	**	**3.3** ± 0.1	4.4 ± 0.2	**	4.2 ± 0.1	**3.2** ± 0.1	***	***	***
% Total titratable acidity	1.2 ± 0.0	**0.8** ± 0.0	**	**1.4** ± 0.0	**1.9** ± 0.0	***	1.3 ± 0.0	1.4 ± 0.0	ns	**1.1** ± 0.4	1.3 ± 0.7	ns	***	***
Soluble solids (°Brix)	**10.7** ± 0.9	**12.5** ± 1.7	**	8.7 ± 1.2	9.8 ± 0.9	**	9.1 ± 0.8	10.1 ± 0.8	**	**8.2** ± 1.2	**9.4** ± 0.9	**	***	***
% Humidity	86.9 ± 0.5	83.6 ± 1.5	***	**98.3** ± 0.4	83.4 ± 2.3	***	**79.2** ± 0.1	**86.1** ± 4.5	***	88.9 ± 0.4	**79.3** ± 0.1	***	***	***
% Ash	2.4 ± 0.5	**3.2** ± 0.4	***	**13.2** ± 0.4	**1.3** ± 0.4	***	6.7 ± 1.6	2.6 ± 0.7	***	**1.5** ± 0.1	2.2 ± 0.7	***	***	***
Maturity index	**10.1** ± 3.2	**12.6** ± 1.3	ns	**6.3** ± 1.4	**5.1** ± 0.5	ns	7.6 ± 2.1	8.1 ± 2.8	ns	7.6 ± 1.6	9.2 ± 4.2	ns	***	***
‘Giant purple’ ecotype (GPE)														
Fruit weight (g)				127.1 ± 16.8	**144.2** ± 20.2	**	**105.7** ± 29.5	**127.6** ± 23.5	ns	**144.4** ± 20.9	135.2 ± 22.3	ns	***	***
Pulp weight (g)				**70.6** ± 13.7	**85.7** ± 14.6	**	**62.4** ± 16.0	**74.6** ± 16.8	ns	63.6 ± 30.8	78.7 ± 16.4	*	ns	**
Seed jelly weight (g)				36.4 ± 7.6	41.6 ± 10.5	*	**28.9** ± 11.4	**43.9** ± 8.6	***	**62.7** ± 20.8	**36.7** ± 12.2	***	***	***
ED (cm)				5.6 ± 0.3	**6.0** ± 0.4	**	**5.3** ± 0.6	**5.7** ± 0.4	*	**5.8** ± 0.3	5.8 ± 0.4	ns	**	***
LD (cm)				7.2 ± 0.6	**7.5** ± 0.2	*	**7.1** ± 0.7	7.1 ± 0.6	ns	**7.9** ± 0.5	**7.0** ± 0.6	***	**	**
pH				**3.4** ± 0.1	**3.5** ± 0.1	**	**4.4** ± 0.2	3.6 ± 0.1	***	4.1 ± 0.2	**4.4** ± 0.2	***	***	***
% Total titratable acidity				**0.8** ± 0.0	**0.6** ± 0.2	ns	1.0 ± 0.2	0.8 ± 0.2	ns	**1.3** ± 0.4	**1.4** ± 0.2	ns	***	***
Soluble solids (°Brix)				**9.9** ± 0.5	**11.1** ± 0.9	***	**8.7** ± 1.2	10.5 ± 2.0	**	9.4 ± 1.2	**10.1** ± 0.7	*	***	*
% Humidity				**80.0** ± 0.6	77.8 ± 1.2	***	**87.8** ± 3.2	**77.5** ± 0.8	ns	85.4 ± 3.0	**84.9** ± 2.4	ns	***	***
% Ash				**3.3** ± 1.1	**3.4** ± 0.6	ns	1.7 ± 0.4	2.8 ± 0.7	***	**0.9** ± 0.4	**1.6** ± 0.7	**	***	***
Maturity index				**13.6** ± 4.1	**18.1** ± 11.2	ns	12.1 ± 2.2	15.7 ± 9.4	ns	**8.9** ± 4.1	**7.5** ± 1.5	ns	***	***

The range is shown in bold. ED: equatorial diameter; LD: longitudinal diameter. Significance of differences between the control and graft (A_CG_); altitude in control (A_AC_); altitude in the graft (A_AG_) is given: ns, not significant, * *p* < 0.1, ** *p* <0.01, *** *p* <0.001.

**Table 5:** Average values of bioactive compounds and antioxidant activity of the ‘giant orange’ tree tomato ecotype (GOE) at different altitudes.

Altitude Range (Masl)			2010–2250			2260–2500			2510–2750			2760–3000			A_AC_	A_AG_
			Control	Grafting	A_CG_	Control	Grafting	A_CG_	Control	Grafting	A_CG_	Control	Grafting	A_CG_		
Vitamin C (mg/100 g DW)		Shell	12.6 ± 0.9	**lnd**	**	11.0 ± 1.3	9.0 ± 0.5	**	**lnd**	**19.2** ± 0.1	***	**20.2** ± 1.4	lnd	**	**	**
		Pulp	18.2 ± 3.6	16.3 ± 3.5	ns	**0.7** ± 0.0	**2.4** ± 0.0	**	6.0 ± 0.8	**32.1** ± 1.3	**	**32.5** ± 1.9	5.8 ± 0.1	**	**	**
		Seed jelly	**186.8** ± 2.4	112.6 ± 1.4	**	**22.4** ± 1.9	**148.1** ± 2.0	**	121.8 ± 9.7	**38.0** ± 2.7	**	42.4 ± 4.8	**148.6** ± 5.7	**	***	***
		Total	**217.6** ± 3.8	129.0 ± 3.6	***	**34.7** ± 0.6	**159.5** ± 1.1	***	127.8 ± 6.6	**89.3** ± 1.0	***	95.1 ± 1.1	154.4 ± 4.1	***	***	***
Organic acid (g/100 g DW)	Citric acid	Shell	**6.9** ± 1.9	5.0 ± 0.7	ns	5.1 ± 0.2	**5.7** ± 0.4	ns	5.8 ± 0.1	**4.7** ± 0.5	ns	**4.7** ± 0.3	5.0 ± 0.1	ns	ns	ns
		Pulp	17.8 ± 1.7	9.0 ± 0.8	*	15.0 ± 1.2	9.25 ± 3.1	*	13.8 ± 1.3	12.4 ± 0.1	ns	20.0 ± 0.2	9.7 ± 0.3	***	*	*
		Seed jelly	9.4 ± 1.9	8.5 ± 0.5	ns	8.8 ± 0.0	6.3 ± 0.3	*	12.1 ± 0.5	10.6 ± 0.8	ns	19.4 ± 1.3	14.9 ± 1.1	*	**	**
	Malic acid	Shell	**0.6** ± 0.1	0.3 ± 0.1	*	0.4 ± 0.0	**0.2** ± 0.0	ns	0.5 ± 0.0	**1.1** ± 0.1	*	**0.3** ± 0.0	0.4 ± 0.0	***	*	**
		Pulp	1.0 ± 0.1	0.6 ± 0.0	*	0.3 ± 0.0	1.4 ± 0.2	*	0.3 ± 0.2	1.2 ± 0.0	*	0.1 ± 0.0	0.7 ± 0.0	***	**	**
		Seed jelly	0.7 ± 0.1	0.8 ± 0.3	ns	0.4 ± 0.0	0.4 ± 0.1	ns	0.4 ± 0.0	1.0 ± 0.1	*	0.9 ± 0.1	0.4 ± 0.0	**	**	**
	Tartaric acid	Shell	7.7 ± 2.0	**5.4** ± 1.0	*	**3.3** ± 0.2	5.7 ± 0.4	*	6.7 ± 0.3	**24.6** ± 2.8	*	10.4 ± 0.2	7.8 ± 0.1	**	***	***
		Pulp	9.9 ± 0.6	3.9 ± 0.3	**	15.9 ± 1.1	27.7 ± 6.6	**	15.9 ± 1.4	18.7 ± 0.3	ns	18.5 ± 0.3	16.5 ± 0.6	*	**	***
		Seed jelly	22.4 ± 1.6	15.0 ± 1.4	*	14.4 ± 0.1	14.2 ± 0.5	ns	17.2 ± 0.6	19.6 ± 1.3	ns	19.7 ± 1.5	15.1 ± 0.7	*	ns	*
	Total		76.4 ± 2.0	**48.4** ± 1.8	**	**63.7** ± 2.5	72.3 ± 4.0	*	72.8 ± 4.0	**93.8** ± 6.0	*	**94.0** ± 2.9	70.3 ± 1.0	**	**	**
Phenolics (mg/100 g DW)	Luteolin	Shell	lnd	121.0 ± 4.21	***	lnd	lnd		438.3 ± 6.5	163.7 ± 1.03	*	**993.3** ± 7.3	652.3 ± 27.2	*	*	***
		Pulp	lnd	lnd		lnd	lnd		lnd	lnd		lnd	lnd		lnd	lnd
		Seed jelly	lnd	lnd		lnd	lnd		lnd	lnd		lnd	lnd		lnd	lnd
	Ferulic acid	Shell	1029.6 ± 21.1	5344.3 ± 110.5	**	687.1 ± 0.1	7830.5 ± 10.9	*	3909.4 ± 60.3	2561.2 ± 32.8	*	**10,792.8** ± 18.6	4112.6 ± 19.9	*	***	*
		Pulp	301.5 ± 38.6	321.7 ± 42.4	ns	338.3 ± 51.0	349.0 ± 17.8	ns	1138.2 ± 150.6	323.9 ± 1.6	*	705.2 ± 24.8	1084.3 ± 39.3	**	**	***
		Seed jelly	lnd	lnd		lnd	lnd		lnd	lnd		266.9 ± 33.7	lnd	***	lnd	lnd
	Caffeic acid	Shell	1233.3 ± 42.3	2764.3 ± 28.6	*	lnd	5411.1 ± 34.1	***	2460.9 ± 39.9	2232.1 ± 19.9	ns	**10,339.9** ± 97.1	2059.1 ± 48.7	**	***	**
		Pulp	83.8 ± 0.6	93.5 ± 8.8	ns	283.2 ± 6.2	416.5 ± 39.4	*	582.7 ± 15.0	lnd	***	490.5 ± 9.6	244.6 ± 23.1	**	**	**
		Seed jelly	lnd	lnd		lnd	lnd		lnd	lnd		lnd	lnd		lnd	lnd
	Total		2648.3 ± 32.4	8645.0 ± 161.2	***	**1308.5** ± 6.4	**14,007.1** ± 165.6	**	8529.5 ± 152.1	**5281.0** ± 59.0	*	**23,588.6** ± 47.1	8152.8 ± 186.1	**	***	**
Carotenoids (mg/100 g DW)	Lutein	Shell	44.9 ± 4.7	33.7 ± 3.3	ns	5.7 ± 0.0	5.4 ± 1.0	ns	33.7 ± 3.1	27.5 ± 7.9	ns	112.9 ± 4.6	7.4 ± 0.7	**	***	**
		Pulp	18.3 ± 3.4	5.5 ± 0.1	*	11.1 ± 2.9	6.9 ± 0.1	ns	6.2 ± 0.2	49.1 ± 11.0	*	**113.6** ± 26.6	**5.2** ± 0.0	*	**	**
		Seed jelly	22.8 ± 2.3	12.5 ± 0.5	*	42.6 ± 4.2	5.8 ± 0.1	**	12.8 ± 0.4	22.6 ± 2.8	*	64.5 ± 17.8	9.4 ± 0.7	*	*	**
	β–cryptoxanthin	Shell	1.8 ± 0.0	1.0 ± 0.0	**	**0.5** ± 0.0	2.6 ± 0.4	*	1.2 ± 0.0	0.8 ± 0.1	*	5.1 ± 0.2	1.5 ± 0.1	**	***	**
		Pulp	4.5 ± 0.0	3.7 ± 0.2	*	3.1 ± 0.0	6.5 ± 0.8	**	2.9 ± 0.2	3.4 ± 0.2	*	9.7 ± 2.4	2.9 ± 0.2	*	*	**
		Seed jelly	6.4 ± 0.2	3.2 ± 0.1	**	0.9 ± 0.0	3.9 ± 0.5	*	3.7 ± 0.3	1.3 ± 0.1	*	**19.5** ± 2.0	1.9 ± 0.3	**	***	**
	β–carotene	Shell	0.6 ± 0.1	0.9 ± 0.1	*	1.0 ± 0.3	**0.4** ± 0.0	*	1.3 ± 0.2	0.6 ± 0.2	*	4.7 ± 1.7	2.1 ± 0.7	ns	*	*
		Pulp	8.8 ± 0.4	9.0 ± 0.7	ns	14.2 ± 2.1	6.5 ± 0.3	*	5.2 ± 1.1	3.5 ± 0.8	*	23.7 ± 3.6	11.3 ± 2.3	*	**	*
		Seed jelly	37.9 ± 2.9	17.8 ± 3.2	*	21.2 ± 0.4	6.4 ± 0.7	**	6.6 ± 1.7	3.1 ± 0.4	*	37.1 ± 1.6	**43.1** ± 7.5	ns	***	**
	Total		145.9 ± 1.6	87.3 ± 0.9	*	100.2 ± 1.1	**44.2** ± 0.4	**	**73.5** ± 0.8	**112.0** ± 2.6	*	**390.7** ± 6.7	84.5 ± 1.4	***	***	*
Antioxidant activity ABTS (µmol TE/g DW)		Shell	70.2 ± 2.5	**109.5** ± 2.1	***	**39.8** ± 4.0	**92.7** ± 2.0	***	**117.4** ± 3.0	99.9 ± 1.1	***	73.5 ± 0.7	**92.7** ± 3.2	***	***	***
		Pulp	30.7 ± 3.0	**4.4** ± 1.1	***	**31.9** ± 1.2	16.4 ± 3.4	***	31.6 ± 5.8	7.0 ± 1.2	***	**7.0** ± 0.9	**16.7** ± 1.7	***	***	***
		Seed jelly	**79.1** ± 0.9	**119.7** ± 1.7	***	40.3 ± 1.0	55.1 ± 2.4	***	**24.2** ± 2.0	21.8 ± 1.1	*	64.5 ± 1.6	**14.5** ± 8.3	***	***	***
Antioxidant activity DPPH (µmol AAE/g DW)		Shell	20.9 ± 1.9	25.0 ± 2.1	*	**4.9** ± 0.3	**26.3** ± 2.2	***	16.9 ± 2.5	**23.7** ± 2.3	**	**31.4** ± 0.3	23.9 ± 3.2	**	***	ns
		Pulp	13.4 ± 0.8	**7.6** ± 1.4	***	10.9 ± 1.5	**18.6** ± 1.8	***	**10.3** ± 2.0	9.0 ± 1.1	ns	**20.5** ± 3.9	10.0 ± 0.7	**	***	***
		Seed jelly	13.2 ± 3.1	**21.2** ± 1.3	**	**9.0** ± 1.2	11.3 ± 0.3	*	**13.8** ± 2.9	**9.9** ± 1.3	*	11.9 ± 0.4	15.2 ± 2.4	*	*	***

The range is shown in bold. Significance of differences between the control and graft (A_CG_); altitude in control (A_AC_); altitude in the graft (A_AG_) is given: ns. not significant, * *p* < 0.1, ** *p* < 0.01, *** *p* < 0.001; lnd, not detected limit; TE, Trolox equivalent; AAE, ascorbic acid equivalent.

**Table 6:** Average values of bioactive compounds and antioxidant activity of the ‘giant purple’ tree tomato ecotype (GPE) at different altitudes.

Altitude Range (Masl)			2260–2500			2510–2750			2760–3000			A_AC_	A_AG_
			Control	Grafting	A_CG_	Control	Grafting	A_CG_	Control	Grafting	A_CG_		
Vitamin C (mg/100 g DW)		Shell	**lnd**	**lnd**		13.9 ± 1.5	**lnd**	***	**22.5** ± 2.5	**21.4** ± 0.1	ns	*	***
		Pulp	30.3 ± 0.3	36.6 ± 0.4	**	**34.7** ± 2.7	**48.6** ± 5.3	*	**0.8** ± 0.1	**29.8** ± 0.5	***	**	**
		Seed jelly	**133.9** ± 11.7	71.3 ± 6.9	*	40.2 ± 0.4	**126.4** ± 7.3	**	**33.4** ± 1.5	**29.3** ± 1.7	ns	**	**
		Total	**164.2** ± 8.5	107.9 ± 4.8	***	88.8 ± 3.5	**175.0** ± 1.5	***	**56.7** ± 0.8	**80.5** ± 1.7	***	***	***
Organic acid (g/100 g DW)	Citric acid	Shell	3.4 ± 0.1	5.2 ± 0.5	*	6.0 ± 0.4	12.8 ± 1.0	*	7.6 ± 0.6	6.5 ± 0.1	ns	**	***
		Pulp	9.1 ± 0.8	14.6 ± 1.9	*	13.4 ± 0.2	9.5 ± 0.5	**	18.8 ± 1.0	23.7 ± 0.0	*	**	**
		Seed jelly	7.3 ± 0.2	5.5 ± 0.8	*	8.2 ± 0.4	5.4 ± 0.2	*	9.5 ± 0.0	12.1 ± 0.9	*	**	**
	Malic acid	Shell	0.3 ± 0.0	0.5 ± 0.0	***	0.5 ± 0.0	0.8 ± 0.0	*	0.3 ± 0.0	0.4 ± 0.0	***	***	**
		Pulp	0.5 ± 0.1	0.9 ± 0.1	*	0.6 ± 0.1	0.7 ± 0.0	ns	0.1 ± 0.0	0.2 ± 0.0	ns	*	*
		Seed jelly	0.6 ± 0.0	0.6 ± 0.1	ns	1.0 ± 0.1	0.5 ± 0.0	*	1.3 ± 0.0	1.4 ± 0.1	ns	**	**
	Tartaric acid	Shell	2.4 ± 0.1	13.5 ± 1.2	**	26.6 ± 1.2	15.4 ± 0.9	**	8.1 ± 0.8	19.6 ± 0.4	**	***	***
		Pulp	5.5 ± 0.4	19.3 ± 4.1	*	15.3 ± 0.2	12.4 ± 0.7	*	17.6 ± 3.8	24.3 ± 0.0	ns	*	*
		Seed jelly	12.9 ± 0.2	16.6 ± 2.1	ns	15.3 ± 0.9	18.9 ± 0.8	*	17.0 ± 0.0	27.6 ± 2.2	*	*	**
	Total		**42.0** ± 0.2	76.7 ± 1.2	*	**87.0** ± 0.4	**76.3** ± 0.5	*	80.2 ± 0.7	**115.8** ± 0.4	**	***	**
Phenolics (mg/100 g DW)	Luteolin	Shell	414.8 ± 72.8	**593.7** ± 43.0	*	131.6 ± 16.9	lnd		58.2 ± 1.4	lnd	***	**	*
		Pulp	lnd	lnd		lnd	lnd		lnd	lnd		lnd	lnd
		Seed jelly	lnd	lnd		lnd	lnd		lnd	lnd		lnd	lnd
	Ferulic acid	Shell	5573.6 ± 67.7	**6017.5** ± 156.8	ns	4005.6 ± 31.4	299.9 ± 56.5	**	2941.3 ± 48.8	1652.6 ± 12.9	**	*	**
		Pulp	1629.3 ± 44.3	1537.4 ± 23.1	ns	252.6 ± 37.1	2270.5 ± 82.5	***	lnd	125.5 ± 8.7	***	*	*
		Seed jelly	lnd	lnd		lnd	lnd		lnd	lnd		ln	lnd
	Caffeic acid	Shell	3499.6 ± 50.8	**4380.6** ± 64.2	ns	2513.2 ± 56.0	38.3 ± 4.6	***	2415.9 ± 38.8	1310.3 ± 68.6	**	ns	***
		Pulp	891.7 ± 24.4	793.8 ± 7.2	ns	118.2 ± 1.2	952.7 ± 82.7	***	lnd	lnd		**	*
		Seed jelly	lnd	lnd		lnd	lnd		lnd	lnd		lnd	lnd
	Total		**12,009.1** ± 216.2	**13,323.0** ± 276.8	ns	7021.3 ± 103.3	3561.4 ± 107.7	**	**5415.5** ± 97.5	**3088.4** ± 23.0	**	*	**
Carotenoids (mg/100 g DW)	Lutein	Shell	27.7 ± 9.4	23.2 ± 5.3	ns	**74.6** ± 7.3	**3.5** ± 0.6	**	15.6 ± 1.1	26.9 ± 1.2	**	**	**
		Pulp	31.8 ± 3.8	10.7 ± 0.6	*	54.0 ± 2.4	21.1 ± 7.7	*	31.9 ± 3.1	19.8 ± 4.0	*	ns	ns
		Seed jelly	63.5 ± 12.6	19.0 ± 1.0	*	37.0 ± 1.7	17.0 ± 1.0	*	73.5 ± 3.1	16.8 ± 3.9	**	*	*
	β–cryptoxanthin	Shell	1.0 ± 0.2	**0.5** ± 0.1	*	0.9 ± 0.1	1.4 ± 0.4	ns	1.8 ± 0.2	1.1 ± 0.1	*	*	*
		Pulp	5.1 ± 0.4	2.9 ± 0.3	*	5.6 ± 0.4	5.6 ± 0.1	ns	6.5 ± 0.3	3.3 ± 0.2	**	*	**
		Seed jelly	5.3 ± 1.0	3.3 ± 0.3	ns	2.5 ± 0.3	5.8 ± 0.4	*	**13.4** ± 1.3	6.4 ± 0.1	*	**	**
	β–carotene	Shell	6.5 ± 1.1	1.3 ± 0.0	*	3.8 ± 0.7	3.3 ± 1.2	ns	1.1 ± 0.0	**0.5** ± 0.1	**	*	*
		Pulp	4.5 ± 0.2	3.7 ± 0.1	*	13.6 ± 2.2	3.8 ± 0.9	*	12.2 ± 0.7	4.3 ± 0.0	**	*	*
		Seed jelly	**17.4** ± 0.1	2.6 ± 0.1	***	16.7 ± 4.9	8.7 ± 2.4	*	9.4 ± 2.2	3.3 ± 0.4	*	*	*
	Total		**162.6** ± 3.2	**67.4** ± 0.9	*	**208.7** ± 5.9	70.2 ± 1.6	*	165.4 ± 1.3	**82.3** ± 1.1	**	*	*
Antioxidant activity ABTS (µmol TE/g DW)		Shell	**137.9** ± 0.9	**137.1** ± 1.6	ns	**70.8** ± 0.9	**10.7** ± 2.2	***	74.9 ± 2.2	59.6 ± 2.9	***	***	***
		Pulp	**18.9** ± 4.6	22.3 ± 0.7	**	**3.4** ± 0.8	**32.8** ± 3.2	***	10.1 ± 1.3	**4.1** ± 2.0	***	***	***
		Seed jelly	**119.8** ± 1.2	**93.2** ± 2.7	***	112.2 ± 1.1	92.2 ± 2.5	***	**105.5** ± 1.2	**91.5** ± 2.2	***	***	***
Antioxidant activity DPPH (µmol AAE/g DW)		Shell	**22.9** ± 1.1	**21.9** ± 2.2	ns	22.3 ± 0.9	**7.6** ± 1.1	***	**19.4** ± 0.9	19.6 ± 1.2	ns	**	***
		Pulp	**14.0** ± 2.0	12.0 ± 2.0	ns	11.5 ± 3.6	**15.6** ± 2.3	*	**7.3** ± 1.8	**8.2** ± 1.1	ns	*	***
		Seed jelly	**47.1** ± 14.6	**45.8** ± 2.8	ns	29.7 ± 1.4	37.1 ± 0.4	***	**19.5** ± 1.9	**18.5** ± 3.2	ns	**	***

The range is shown in bold. Significance of differences between the control and graft (A_CG_); altitude in control (A_AC_); altitude in the graft (A_AG_) is given: ns. not significant, * *p* < 0.1, ** *p* < 0.01, *** *p* < 0.001; lnd, not detected limit; TE, Trolox equivalent; AAE, ascorbic acid equivalent.

**Table 7:** Analysis of variance (multifactor ANOVA) considering altitude, ecotype, control and grafting, and part of the fruit.

	2010–2250	2260–2500	2510–2750	2760–3000	*P*_H_	‘Giant Orange’	‘Giant Purple’	*P*_E_	Control	Grafting	*P*_CG_	Shell	Pulp	Seed Jelly	*P*_PF_
ED					***			***			***				
LD					***			ns			ns				
FW					ns			ns			**				
H					ns			ns			ns				
A					***			***			ns				
L*					***			ns			***				
h_ab_					***			ns			ns				
C*_ab_					***			***			ns				
SS					***			ns			***				
pH					***			***			*				
TTA					***			***			ns				
MI					***			***			**				
VC					ns			ns			ns				
CA					**			ns			ns				***
MA					***			**			***				***
TA					***			ns			**				***
FA					ns			ns			ns				***
CAc					**			ns			***				***
Ze					ns			ns			**				***
BC					ns			ns			*				***
BCa					**			*			***				***

Note: ED: equatorial diameter; LD: longitudinal diameter; FW: fruit weight; H: humidity; A: ash; SS: soluble solids; TTA: total titratable acidity; MI: maturity index; VC: vitamin C; CA: Citric acid; MA: malic acid; TA: tartaric acid; Lu: luteolin; FA: ferulic acid; CAc: caffeic acid; Ze: zeaxanthin; BC: β–cryptoxanthin; BCa: β-carotene. Significance of differences in altitude (*P*_H_), ecotype (*P*_E_), control and grafting (*P*_CG_) and part of the fruit (*P*_PF_) is given: ns, not significant, * *p* < 0.1, ** *p* < 0.01, *** *p* < 0.001, and mean separation is shown with coloured pictures, thus, a, yellow; ab, brown; b, grey; bc, lead; c, white.

## Data Availability

The data used to support the findings of this study can be made available by the corresponding author upon request.
